# Functional Genetic Diversity among *Mycobacterium tuberculosis* Complex Clinical Isolates: Delineation of Conserved Core and Lineage-Specific Transcriptomes during Intracellular Survival

**DOI:** 10.1371/journal.ppat.1000988

**Published:** 2010-07-08

**Authors:** Susanne Homolka, Stefan Niemann, David G. Russell, Kyle H. Rohde

**Affiliations:** 1 Molecular Mycobacteriology, Research Center Borstel, Borstel, Germany; 2 Department of Microbiology and Immunology, College of Veterinary Medicine, Cornell University, Ithaca, New York, United States of America; University of New Mexico, United States of America

## Abstract

Tuberculosis exerts a tremendous burden on global health, with ∼9 million new infections and ∼2 million deaths annually. The *Mycobacterium tuberculosis* complex (MTC) was initially regarded as a highly homogeneous population; however, recent data suggest the causative agents of tuberculosis are more genetically and functionally diverse than appreciated previously. The impact of this natural variation on the virulence and clinical manifestations of the pathogen remains largely unknown. This report examines the effect of genetic diversity among MTC clinical isolates on global gene expression and survival within macrophages. We discovered lineage-specific transcription patterns *in vitro* and distinct intracellular growth profiles associated with specific responses to host-derived environmental cues. Strain comparisons also facilitated delineation of a core intracellular transcriptome, including genes with highly conserved regulation across the global panel of clinical isolates. This study affords new insights into the genetic information that *M. tuberculosis* has conserved under selective pressure during its long-term interactions with its human host.

## Author Summary

Genetic polymorphisms among bacterial pathogens are exploited by epidemiologists to monitor disease outbreaks and transmission of specific strains. Genetic variations occur under selective pressures imposed by the human host, leading to variation amongst clinical isolates. Tuberculosis (TB) is an ancient disease caused by *Mycobacterium tuberculosis* that still exerts a tremendous burden on global health. *M. tuberculosis* was widely regarded as a homogeneous population with limited genetic variability. However, data from recent studies suggest that the functional diversity of this pathogen has been underestimated. This study features a panel of isolates spanning the phylogenetic diversity of the *M. tuberculosis* complex. Using microarrays, we found that the genetic diversity of *M. tuberculosis* strains has significant impact on global gene expression. The distinct abilities of strains to survive inside macrophages and alter gene expression during infection highlight novel strategies of intracellular adaptation. We also delineated a core intracellular transcriptome across the panel of strains. This affords new insights into the genetic information that *M. tuberculosis* conserves to remain a successful intracellular pathogen. Elucidating the functional significance of *M. tuberculosis* genetic diversity is important for understanding the diverse outcomes of infection, mechanisms of pathogenesis, and for the development of effective new diagnostic and therapeutic tools.

## Introduction

*Mycobacterium tuberculosis* represents a unique opportunity to explore the impact of evolution and genetic diversity on bacterial host adaptation and pathogenesis. Tuberculosis is an ancient disease, with evidence that the causative agent *M. tuberculosis* has been co-evolving with its human host since mankind emerged from Africa 50,000 years ago [1], [2]. Despite intense efforts, the disease remains a major burden on global health and a strong selective pressure on human evolution [3]. *M. tuberculosis* is the most prominent member of the *Mycobacterium tuberculosis* complex (MTC), comprised of seven closely related species with distinct host tropisms including the human pathogens *M. africanum* and *M. canettii* and the animal adapted species *M. bovis* (bovine), *M. caprae* (goats), *M. pinnipedii* (seals), and *M. microti* (rodents). Members of the MTC are considered genetically monomorphic with a high level of genomic sequence similarity (>99.95%), limited horizontal gene transfer, and a clonal population structure [4], [5]. This apparent homogeneity led to the assumption that genetic diversity among MTC strains would not be of clinical significance. However, recent data based on molecular genotyping methods (i.e. IS*6110* RFLP, spoligotyping, MIRU-VNTR typing) revealed a highly diverse population structure with at least six major geographically-associated lineages that can be further subdivided into well-defined genotypes [1], [2]. Based on multilocus sequence analysis of 89 genes in 108 strains, Hershberg *et al.* conservatively estimated that an average pair of MTC strains would have ∼300 functional differences due to SNPs alone [1]. Thus, it is becoming apparent that the genetic diversity of the MTC has been underestimated.

There is mounting evidence that the genetic variability among clinical isolates may have dramatic consequences on the outcome of infections. For example, studies *in vitro* [6], [7], [8], in animal models [9], [10], [11], [12], [13], and human epidemiology studies [14] have demonstrated strain-dependent variation in key aspects of virulence such as stress survival, transmission, pathology, and lethality. It is also believed that the genetic diversity of the MTC contributes to the wide spectrum of TB clinical presentations, including acute primary TB (localized or disseminated), latent disease and reactivation [15], [16]. However, the molecular mechanisms for such variations in *M. tuberculosis* pathogenesis and host adaptation have been identified in only a few cases. In one example, the hypervirulence of some Beijing strains has been linked to the production of immune modulatory phenolic glycolipid (PGL), whereas a 7bp deletion in the *pks 15/1* gene disables PGL synthesis in many isolates [13], [17], [18]. Genetic variation may also lead to altered metabolism and gene expression pathways important for MTC pathogenesis [19], [20], [21].

A hallmark of pathogenic mycobacteria is the ability to survive within the phagosome of professional phagocytes and persist within pulmonary granulomas for decades. This most intimate of inter-species associations has led to the evolution of strategies to resist antimicrobial effectors and subvert the immune response. Information on the “common” and “variable” responses of clinical isolates of different phylogenetic lineages to host macrophages is crucial for identification of microbial factors required for intracellular survival or involved in the adaptation to specific hosts. However, studies analyzing gene expression and virulence of MTC in infection models have largely been restricted to a limited number of individual strains [22], [23].

This study details a systematic approach to define the degree to which diversity, hard-wired into the bacterial genome, translates into functional phenotypes reflected in expression profiling and bacterial survival within macrophages. We investigated a panel of 17 strains that represent the global phylogeographic diversity of MTC: two reference strains H37Rv and CDC1551 and 3 clinical isolates each from 5 distinct genotypes. The varied survival characteristics of the clinical isolates within macrophages, in addition to the heterogeneity of the *in vitro* and intracellular transcriptome profiles are evidence of the powerful effect genetic variation exerts on global gene expression and host interactions. Our approach of linking well-characterized genotypes to transcriptional responses induced by conditions relevant to infection (*in vitro*, versus resting and activated murine macrophages) is a crucial step toward understanding the functional evolution of MTC and the varied pathologies of tuberculosis.

## Results

### Evolution of MTC clinical isolates evident in basal *in vitro* transcriptome

**Figure 1:**
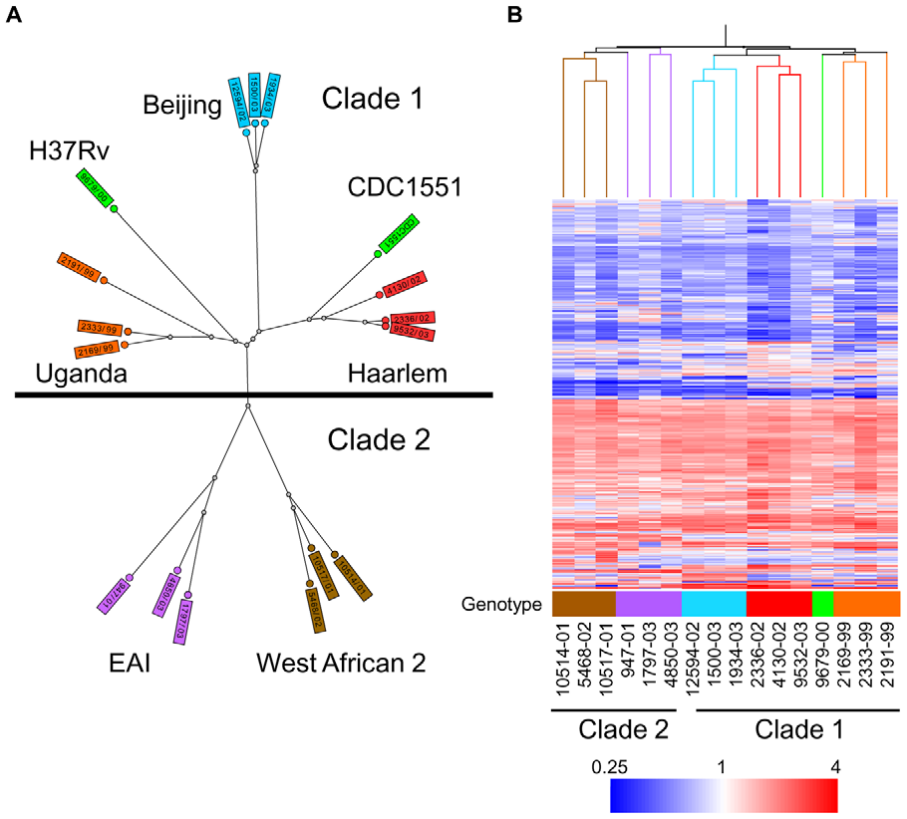
Genetic and transcriptome diversity of *M. tuberculosis* complex (MTC) clinical isolates. (A) Radial neighbor-joining tree based on 24 loci MIRU-VNTR and 43 spacer spoligotyping showing the phylogenetic relationship of strains in this study. Three strains each from the 5 distinct lineages pathogenic to humans plus two sequenced reference strains (H37Rv and CDC1551) were chosen to represent the global diversity of MTC. The color used to denote each genotype is maintained in all subsequent figures for clarity. (B) Condition tree of MTC clinical isolate transcription profiles *in vitro* during log phase growth in 7H9 medium relative to CDC1551 reference strain (three biological replicates). Expression profiles for genes passing quality filters (flagged as present in 42 of 48 samples) with differential expression in at least one strain (up or down >1.5× in at least 1 of 16) were clustered using the Spearman correlation. Each column represents the global transcription profile of a single strain. Genes were clustered vertically based on the distance measure. Red and blue indicate higher or lower gene expression than CDC1551 control, respectively. Unless otherwise indicated, the color scale for expression (4-fold up or down) was used for all subsequent figures.

To determine the functional impact of the natural genetic diversity of pathogenic mycobacteria, we first studied the growth and gene expression of MTC clinical isolates from five global phylogeographical lineages in liquid culture (Figure 1A, Table 1, Supplementary Figure S1). The growth kinetics of most strains in rich medium, especially members of the Beijing, EAI, and West African 2 genotypes, were not significantly different from the reference strains CDC1551 and H37Rv. However, clinical isolates of the Haarlem and Uganda genotypes exhibited slower growth rates *in vitro* (Supplementary Figure S2 and S7A). Microarray profiling of log phase bacteria (OD∼0.6) yielded expression patterns that mirror the phylogenetic relationship of strains as defined by molecular typing methods (Figure 1B). Condition tree analysis of *in vitro* transcriptome data segregated strains into two main branches consistent with the recently described classification of “modern” clade 1 and “ancient” clade 2 strains (Figure 1B) [2]. A total of 156 genes exhibited clade specific profiles (Supplementary Figure S3, Supplementary Tables S1 and S2), highlighting the functional divergence of these two MTC lineages. Gene clusters with higher expression in clade 1 (i.e. *narGHJI*, nitrate reductase operon) or clade 2 (i.e. *mce4* locus, implicated in cholesterol utilization [24]), confirmed by qRT-PCR analysis of *narG* (Supplementary Figure S10A) and *mce4C* (Supplementary Figure S9A) could have a significant impact on the “success” of these strains across their human host populations.

**Table 1:** *Mycobacterium tuberculosis* complex (MTC) clinical isolates investigated in this study.

species	No	lineage	genotype	RD9	TbD1	pks15/1	INH	RMP	PZA	EMB	SM
*M. tuberculosis*	9532/03	Euro-American	Haarlem	+	−	−	s	s	s	s	s
	2336/02	Euro-American	Haarlem	+	−	−	s	s	s	s	s
	4130/02	Euro-American	Haarlem	+	−	−	s	s	s	s	s
	2333/99	Euro-American	Uganda	+	−	−	s	s	s	s	s
	2169/99	Euro-American	Uganda	+	−	−	s	r	s	s	s
	2191/99	Euro-American	Uganda	+	−	−	s	s	s	s	s
	1934/03	East Asian	Beijing	+	−	+	s	s	s	s	s
	1500/03	East Asian	Beijing	+	−	+	s	s	s	s	s
	12594/02	East Asian	Beijing	+	−	+	s	s	s	s	s
	4850/03	Indo Oceanic	EAI	+	+	+	s	s	s	s	s
	1797/03	Indo Oceanic	EAI	+	+	+	s	s	s	s	s
	947/01	Indo Oceanic	EAI	+	+	+	s	s	s	s	s
*M. africanum*	10517/01	West-African 2	WA2	−	+	−	s	s	s	s	s
	5468/02	West-African 2	WA2	−	+	−	s	s	s	s	s
	10514/02	West-African 2	WA2	−	+	−	r	s	s	s	s

Molecular genotyping and drug sensitivity testing of *Mycobacterium tuberculosis* complex (MTC) clinical isolates investigated in this study. Strain lineages are based on phylogenetic analysis by Gagneux *et al.* [86]. TbD1, RD9 and *pks15/1* denote the presence or absence of previously reported phylogenetic markers [87], [88]. The Euro-American strains (Haarlem and Uganda genotypes) contain a 7bp deletion and W. African 2 strains contain a 6bp deletion in *pks15/1*. Abbreviations: EAI (East African Indian), WA2 (West African 2), INH (isoniazid), RMP (rifampicin), EMB (ethambutol), PZA (pyrazinamide), SM (streptomycin), s (sensitive), r (resistant).

**Figure 2:**
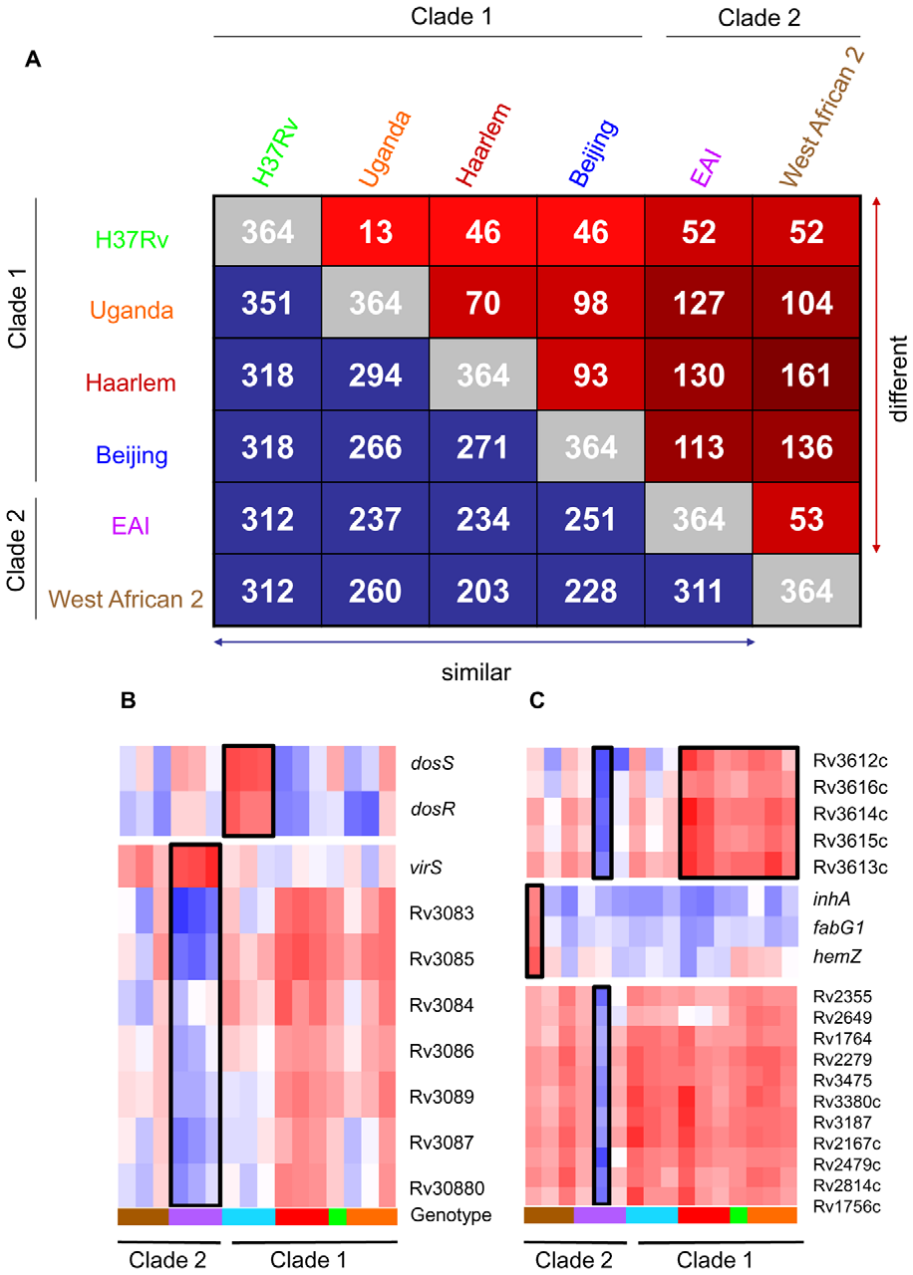
Identification of genotype- and strain- specific *in vitro* transcription profiles. (A) Pair-wise comparison of genes with significant genotype-dependent expression. Includes genes identified by one-way ANOVA of quality-filtered geneset (present in 42 of 48 samples) using Benjamini and Hochberg False Discovery Rate (p<0.01). The matrix of intragenotype comparisons was generated by Tukey post hoc test. The numbers within red squares indicate genes with unique expression patterns between the two intersecting genotypes. (B) Gene tree of select virulence-associate transcriptional regulators with genotype-specific expression signatures. The *dosRS* two-component regulator, overexpressed in Beijing strains, controls the expression of ∼50 genes in response to multiple signals including nitric oxide, hypoxia, and carbon monoxide and is required for infection in animal models [20], [36], [70], [80], [81], [82], [83]. VirS is overexpressed in EAI strains with concomitant decreased transcript levels of the *mymA* operon (Rv3083-Rv3089). *virS* and *mymA* genes play a role in cell wall ultrastructure and are required for growth in activated macrophages and in mouse spleen [52], [53]. (C) Gene tree of select loci exhibiting strain-specific expression and profiles shared across multiple genotypes. Expression ratios indicated by color gradient are *in vitro* log phase transcript levels relative to CDC1551 reference strain (see Fig. 1B for color key).

We identified 364 genes (∼10% of all genes) with significant expression differences (p<0.01) between strains of different genotypes in at least one pair-wise comparison (Figure 2A). Our analyses delineated genotype-specific signatures (Figure 2B) like the *virS-mym* operon (Rv3082c-3089) dysegulated in all EAI strains and the *dosRS* two-component regulator (Rv3132c-3133c) overexpressed in all Beijing strains (Figure 2B). Semi-quantitative qRT-PCR verified the ∼10-fold higher basal expression level of *dosR* in Beijing isolates (Supplementary Figure S8 and S9B), the elevated transcription of *virS* (3-fold) and concomitant repression (∼50-fold) of Rv3083 in EAI strains (Supplementary Figure S8 and S9C). Additionally, we identified profiles shared between genotypes such as the *moa* operon (*moaE3*, *moaC3*, *gphA*, MT3427, MT3427.1) hyper-expressed in Beijing and Haarlem strains. Similarly, transcripts of the Rv3612c-3616c operon, involved in secretion of the virulence factor ESAT-6, were more abundant in the Haarlem and Uganda genotypes while exhibiting diminished expression in EAI strain 1797/03 (Figure 2C). Subsequent 1-way ANOVA analysis (p<0.01) identified a total of 195 genes with strain-specific gene expression profiles (Supplementary Figure S4). The elevated expression of the *fabG1-inhA-hemZ* operon (Rv1483-1485) in a single West African 2 strain, for example, highlights the potential for genetic variation to alter a drug target (InhA) involved in cell wall biosynthesis (Figure 2C). In fact, subsequent analysis of strain 10514/01 revealed a previously described C→T substitution at position −15 upstream of this operon [25] accompanied by isoniazid resistance (data not shown). The negligible detection of eleven *IS6110* related genes in strain 1797/03, defined as a subgroup of the EAI genotype by unique *IS6110* RFLP fingerprinting, illustrates the potential to identify possible deletions among clinical isolates based on gene expression data (Figure 2C, Supplementary Figure S1).

This remarkable degree of diversity in the basal transcriptome of MTC clinical isolates *in vitro* provides compelling evidence that genomic variation among strains results in a greater functional impact than appreciated previously.

### Variable survival of MTC clinical isolates in murine macrophages

**Figure 3:**
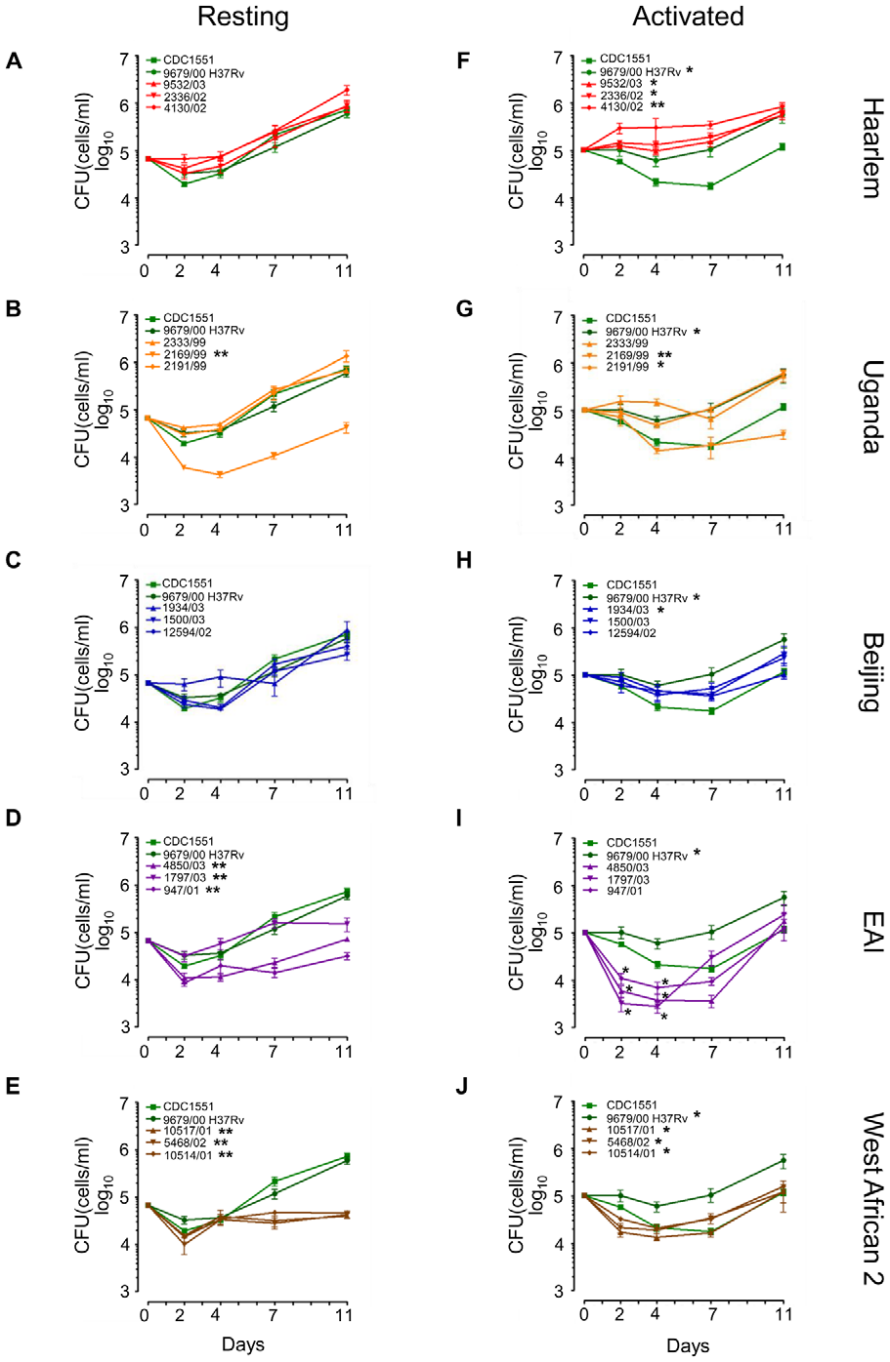
Long-term survival and growth of MTC clinical isolates in macrophages. Resting (A–E) and IFN-γ + LPS activated (F–J) murine bone-marrow derived macrophages were infected at low MOI (∼1∶1) with MTC strains. Quantification of viable CFU at day 0,2,4,7, and 11 post-infection were conducted by lysis of monolayers, serial dilution, and plating on 7H10 medium. Error bars indicate standard error of the mean from two independent biological replicates each consisting of three technical replicates per strain (total of 6 wells/strain). Growth profiles of reference strains, H37Rv and CDC1551, are shown in green in all panels. Asterisks in the legend indicate strains determined to exhibit growth profiles significantly different compared to CDC1551 by ANOVA (* = p<0.05, ** = p<0.001). Although EAI strains did not reach p<0.05 when time was modeled as a continuous variable, modeling time as a nominal variable yielded differences of statistical significance at specific time points, indicated by asterisks at day 2 and day 4 in (I).

To test the hypothesis that the disparate patterns of gene expression described above would influence the ability of MTC strains to survive in their intracellular niche, we analyzed the growth of our panel of 15 clinical isolates and reference strains CDC1551 and H37Rv in resting and activated murine macrophages for 11 days. We found that intracellular growth revealed clade-, genotype- and strain-specific fitness profiles for survival within macrophages even more clearly than *in vitro* growth (Figure 3, Supplementary Figure S7B–C).

In resting macrophages, intracellular survival of most clade 1 clinical isolates was similar to CDC1551 and H37Rv (Figure 3A–C, Supplementary Figure S7B). This was not surprising given that molecular genotyping indicates CDC1551 and H37Rv are related to the Haarlem and Uganda genotypes, respectively (Figure 1A). Consistent with Theus *et al.* [26], we did not observe the previously reported hypervirulence of Beijing strains in our infection model [27], [28] (Figure 3C). It is notable that most clade 2 strains exhibited either an early survival defect (EAI 4850/03, 947/01) and/or significantly impaired replication in resting macrophages compared to clade 1 or reference strains (Figure 3D–E, Supplementary Figure S7B). A single clade 1 isolate within the Uganda genotype (2169/99) was severely attenuated for intramacrophage survival during early stages of infection perhaps linked to its poor *in vitro* replication (Figure 3B, Supplementary Figure S2 and S7B). However, overall there was no direct correlation between *in vitro* growth rates and intracellular fitness. For example, EAI and West African 2 isolates were clearly impaired for survival in resting macrophages compared to Beijing strains, despite similar replication kinetics *in vitro*.

Activation of the host macrophages with interferon-g and LPS accentuated the differences in intracellular growth between clinical isolates (Figure 3F–J). Note the divergence of CDC1551 and H37Rv intracellular growth profiles compared to resting macrophages (Figure 3A and 3F). Although activated macrophages were more effective at suppressing mycobacterial growth until day 7, we did note a late rebound by most MTC isolates after day 7 perhaps indicating delayed bacterial adaptation or a waning of antimicrobial effectors in the activated macrophages. Pair-wise comparisons between all strains using the Tukey-Kramer HSD test delineated significant genotype- and strain-dependent intracellular growth profiles (Supplementary Figure S7C). For example, the Haarlem genotype proved to be better adapted for phagosome survival than Beijing, EAI, or West African 2 isolates (Figure 3F, Supplementary Figure S7C). Contrary to previous reports of enhanced intracellular replication [9], [27], [28], Beijing isolates grew only slightly better than CDC1551 (Figure 3H). Uganda strain 2169/99 survived but failed to grow in activated macrophages, consistent with its subpar growth *in vitro* and in resting macrophages (Figure 3G). In activated macrophages, the growth profiles of clade 2 isolates were distinct from Haarlem, Uganda (except 2169/99), and H37Rv (Supplementary Figure S7C). Strains of the EAI genotype presented a unique profile, experiencing an acute early decline in CFU followed by a rebound to CDC1551 levels by day 11. Intriguingly, West African 2 strains (*M. africanum*) appear better adapted for growth in activated versus resting macrophages (compare Figure 3E and 3J).

### MTC strains exhibit a highly conserved core intracellular transcriptome

**Figure 4:**
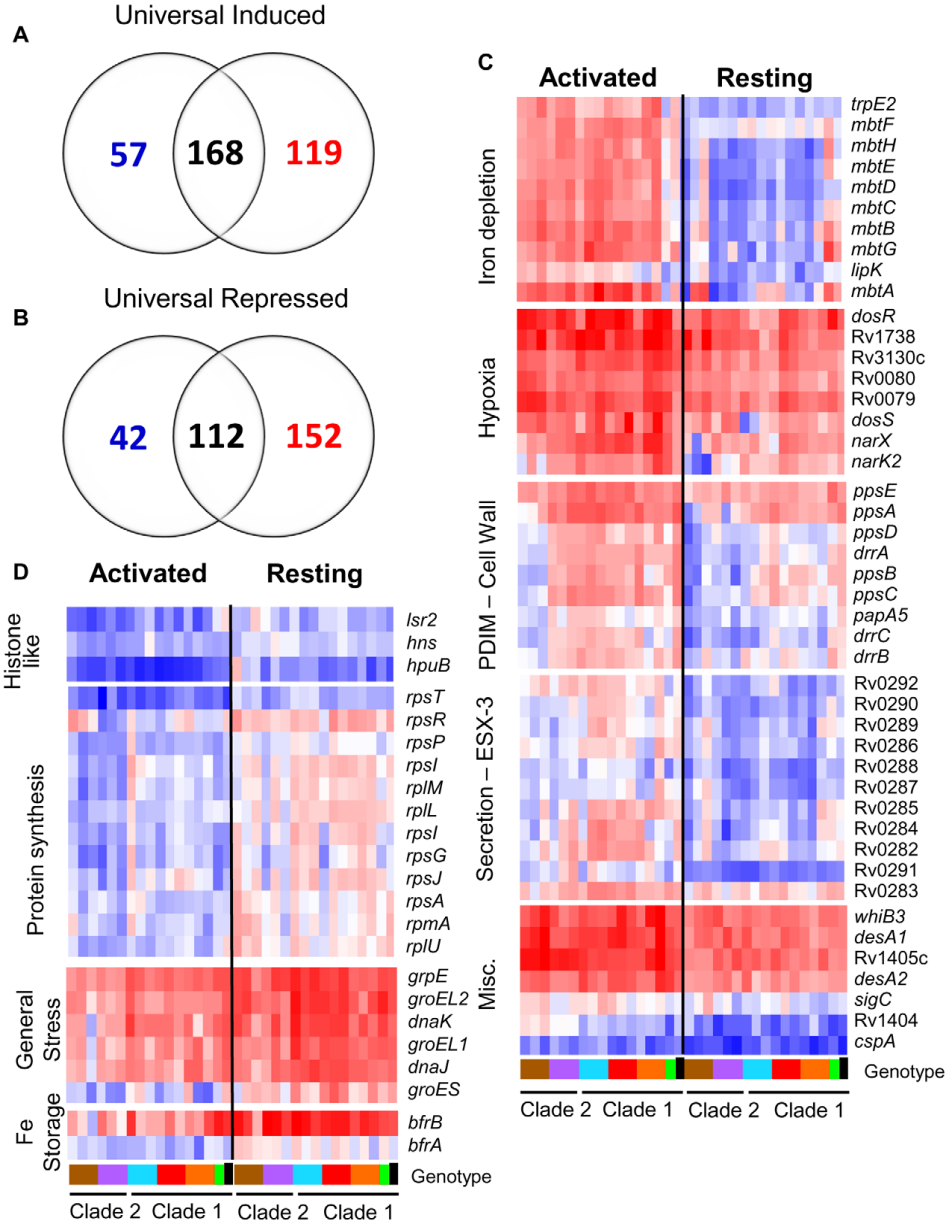
The MTC universal intracellular transcriptome: identification of genes with conserved expression and regulation inside macrophage phagosome across global panel of MTC clinical isolates. Global gene expression profiles of 17 MTC strains 24h post-infection of resting and activated macrophages were determined by microarray. Normalized expression ratios for each strain were determined by comparison of RNA from intracellular bacteria versus control bacteria of the same strain treated identically except for phagocytosis by macrophages. This serves to identify relative responses to phagosomal cues rather than inherent strain-dependent differences in gene expression. (A–B) Venn diagrams showing activation-dependent (red = activated, blue = resting) and independent (black) genes with conserved expression patterns across clinical isolates. “Universal genes” were selected based on trending up or down in all strains (up or down >1.2-fold in 15 of 17 strains) and significant induction or repression in >50% of strains (up or down >1.5× in 8 of 17 strains) in each macrophage type. The starting gene list for this analysis included only genes flagged as present in the majority of samples from both macrophage types. (C) Select genes with higher expression levels in activated versus resting macrophages conserved across all or most clinical isolates. Represents subset of activation-dependent genes identified by one-way ANOVA analysis of all intracellular transcription profiles (Benjamini and Hochberg False Discovery Rate p<0.01). (D) Select genes with higher expression in resting versus activated macrophages. See (C) above for analysis description. Refer to Fig 1B for genotype color bar definition (black box denotes reference strain CDC1551).

To determine the effect of natural genomic variation on the response of MTC isolates to the intracellular niche, we conducted microarray analysis of intracellular MTC bacilli 24h after infection of resting and activated murine macrophages. This facilitated identification of a highly-conserved, core intramacrophage transcriptome which included 280 genes either universally induced (168 genes) or repressed (112 genes) in all strains, regardless of activation state of the host cell (Figure 4A–B, Supplementary Figure S5, Supplementary Tables S3, S4, S5, S6, S7 and S8). The induction of genes with roles in hypoxia, oxidative and nitrosative stress, cell wall remodeling, regulation, and fatty acid metabolism across the panel of clinical isolates confirms previous studies using single strains [22], [23] and affirms their association with the adoption of an intracellular lifestyle (Supplementary Figure S5A, Supplementary Tables S3, S4 and S5). The 112 universally repressed genes, in addition to reflecting suppression of growth and energy metabolism, indicate modulation of cell wall composition (*acpM*, *kasA*, *fabD*, lipoproteins), lipid and cholesterol transport (*mmpL9*, *mmpL12*, *mce1* genes *lprK*, Rv0167, Rv0170, Rv0172, Rv0176, and *mce4* genes Rv3492c, Rv3493c, Rv3497c) [29], [30], and transcriptional regulation (*sigD*, *cspA*, Rv3676) (Supplementary Figure S5B, Supplementary Tables S6, S7 and S8). Approximately 40% (114/280) of the “universal intracellular transcriptome” that exhibited conserved regulation among clinical isolates were genes of unknown function. The core intracellular transcriptome highlights fundamental stress responses, pathways, and genes involved in intracellular survival that are conserved across the MTC.

Following the initial encounter of pathogenic mycobacteria with resting alveolar macrophages, the onset of the adaptive immune response presents an enhanced “stress test” for MTC. Classical activation of macrophages by IFN-γ constrains bacterial growth by overriding the block in phagolysosome fusion and upregulating antimicrobial effectors, such as ROI and RNI [31]. However, despite controlling the infection, the failure to eradicate MTC indicates that the pathogen is capable of adapting to or modulating this environment to survive. To identify transcriptional responses that correlated with activation status of the macrophage, we used 1-way ANOVA analysis of intracellular array data to identify 719 genes whose expression was significantly influenced by macrophage activation (p<0.01) across the panel of strains.

Responses to known phagosome parameters enhanced by activation, such as nitric oxide production (*dosR* regulon) and iron limitation (mycobactin operon), were evident among the 317 genes with elevated induction in activated macrophages (Figure 4C, Supplementary Table S10). The conserved induction of certain transcriptional regulators (i.e. *whiB3*, *cpsA*), transporters (i.e.ESX-3), lipid metabolism enzymes (i.e. *desA1*, *desA2*, *ppsABCDE*), and 109 genes of unknown function in all 15 clinical MTC isolates within activated macrophages indicates a role for these loci in survival under immune pressure (Figure 4C, Supplementary Table S9).

Our analyses also delineated 402 genes with higher induction ratios in resting versus activated macrophages (Figure 4D, Supplementary Table S10). Many genes, such as *rps* and *rpl* genes encoding ribosomal proteins involved in protein synthesis and histone-like proteins (*lsr2*, *hns*, *hupB*) also fell into this category because they were highly repressed in activated macrophages (Figure 4D). The enhanced expression of iron storage genes *bfrA* and *bfrB* are indicative of the iron-replete environment of the transferrin-accessible resting phagosome [32]. Interestingly, markers of the general stress response (*grpE*, *groEL*, *groEL2*, *groES*) were induced more highly by MTC strains in resting macrophages. This would imply that these chaperones are required to sustain growth because many of the genes that exhibited higher expression in resting macrophages are clearly linked to bacterial growth and nutrient acquisition consistent with replicating organisms.

### Lineage specific transcriptional responses to intracellular cues

**Figure 5:**
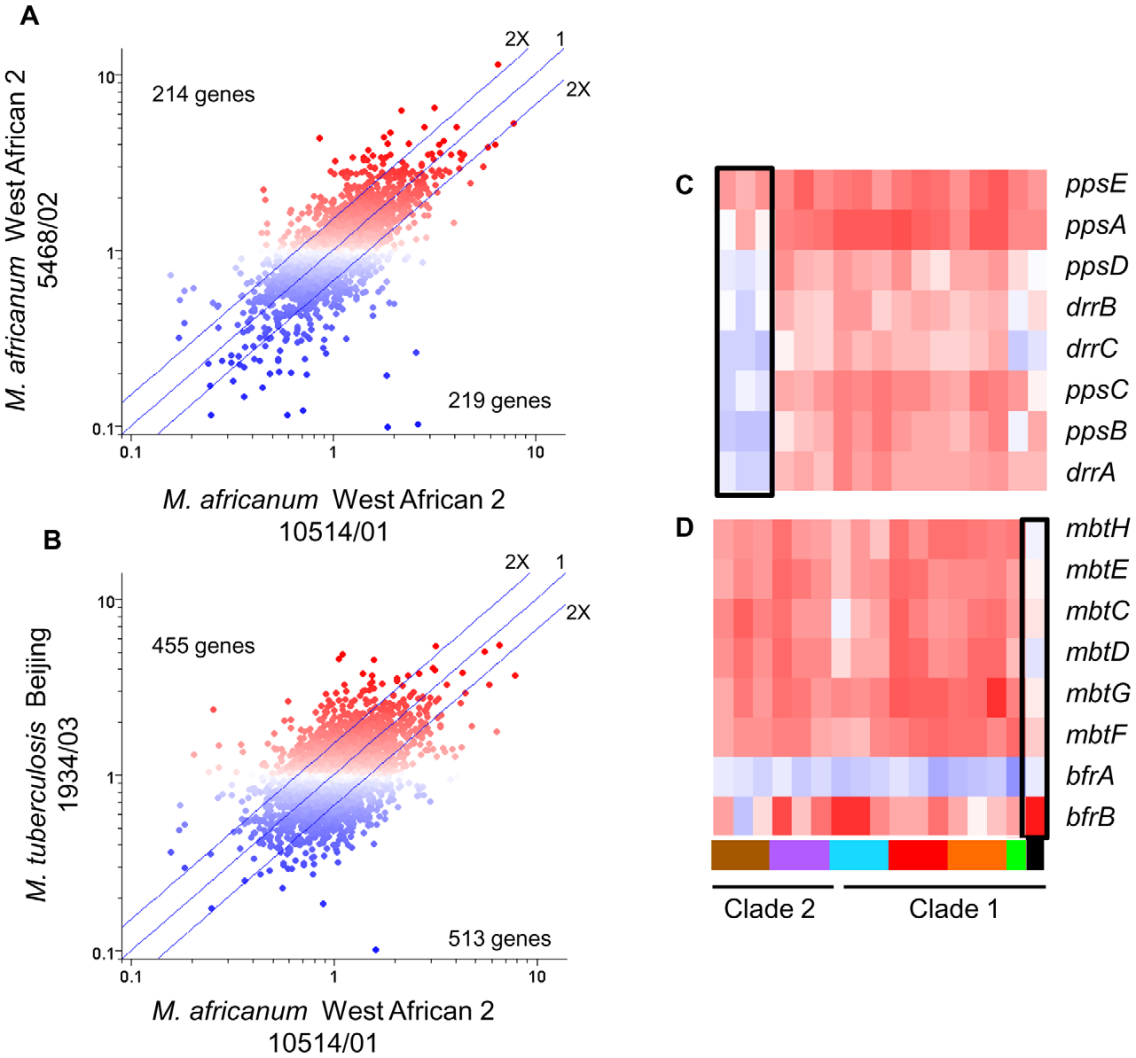
Lineage-specific transcriptional responses of MTC clinical isolates to macrophage invasion. (A) Intragenotype comparison of gene expression profiles 24h post-infection of resting macrophages. Scatter plot showing similarity of transcriptome modulation of two *M. africanum* (genotype West African 2) strains 10514/01 (x-axis) versus 5468/02 (y-axis). (B) Intergenotype comparison of gene expression profiles 24hr post-infection of resting macrophages. Scatter plot showing *M. africanum*, genotype West African 2 strain 10514/01 (x-axis) versus *M. tuberculosis* Beijing strain 1934/03 (y-axis). Each spot represents a single gene with expression ratios >1 indicating intracellular induction (red spots) and <1 indicating intracellular repression (blue spots). The degree of similarity in the regulation of a gene between two strains is shown by proximity to the middle diagonal line (ratio = 1). Axes show normalized expression ratio relative to strain-matched extracellular control in a log_10_ scale. The number of genes with >2-fold differences in intracellular regulation are indicated on the graph. The increased scatter and number of differentially regulated genes seen in strains of different genotypes (B) reflects the diversity of responses to the vacuole environment observed in strains from genetically diverse lineages. Gene trees showing examples genotype- (C), and strain- (D) specific gene regulation after 24h infection of activated macrophages. Refer to Fig 1B for genotype color bar definition (black box denotes reference strain CDC1551).

The disparate abilities of MTC clinical isolates to survive and replicate in macrophages could be attributable to both the distinct baseline patterns of *in vitro* gene expression (described above) and/or to strain-dependent transcriptional adaptation to phagosome-derived cues. Intracellular transcriptome data, which were normalized to strain-matched extracellular controls to minimize the contribution of *in vitro* gene expression differences, revealed that MTC clinical isolates also exhibited distinct responses to environmental cues within the phagosome. Although the responses were more divergent than observed from *in vitro* microarrays, the changes in gene expression induced by macrophage invasion still reflected the phylogenetic relationship of strains with greater correlation between intragenotypic (Figure 5A) versus intergenotypic (Figure 5B) profiles. One-way ANOVA analyses (p<0.01) identified 625 genes with genotype-specific and 293 genes with strain-specific expression across both resting and activated macrophage datasets. Similar analysis of each macrophage state separately revealed 114 and 266 genes with genotype-specific expression patterns in resting and activated macrophages, respectively. The failure of *M. africanum* (West African 2) strains to induce the phthiocerol dimycocerosate (PDIM) locus, a complex cell wall lipid unique to mycobacteria, exemplifies the genotype-specific regulation of virulence factors revealed by this study (Figure 5C, Supplementary Figure S10B). The unique responses of individual strains (Figure 5D) provided further evidence of the impact of natural genetic variation on environmental sensing and phagosome adaptation across the panel of MTC clinical isolates.

**Figure 6:**
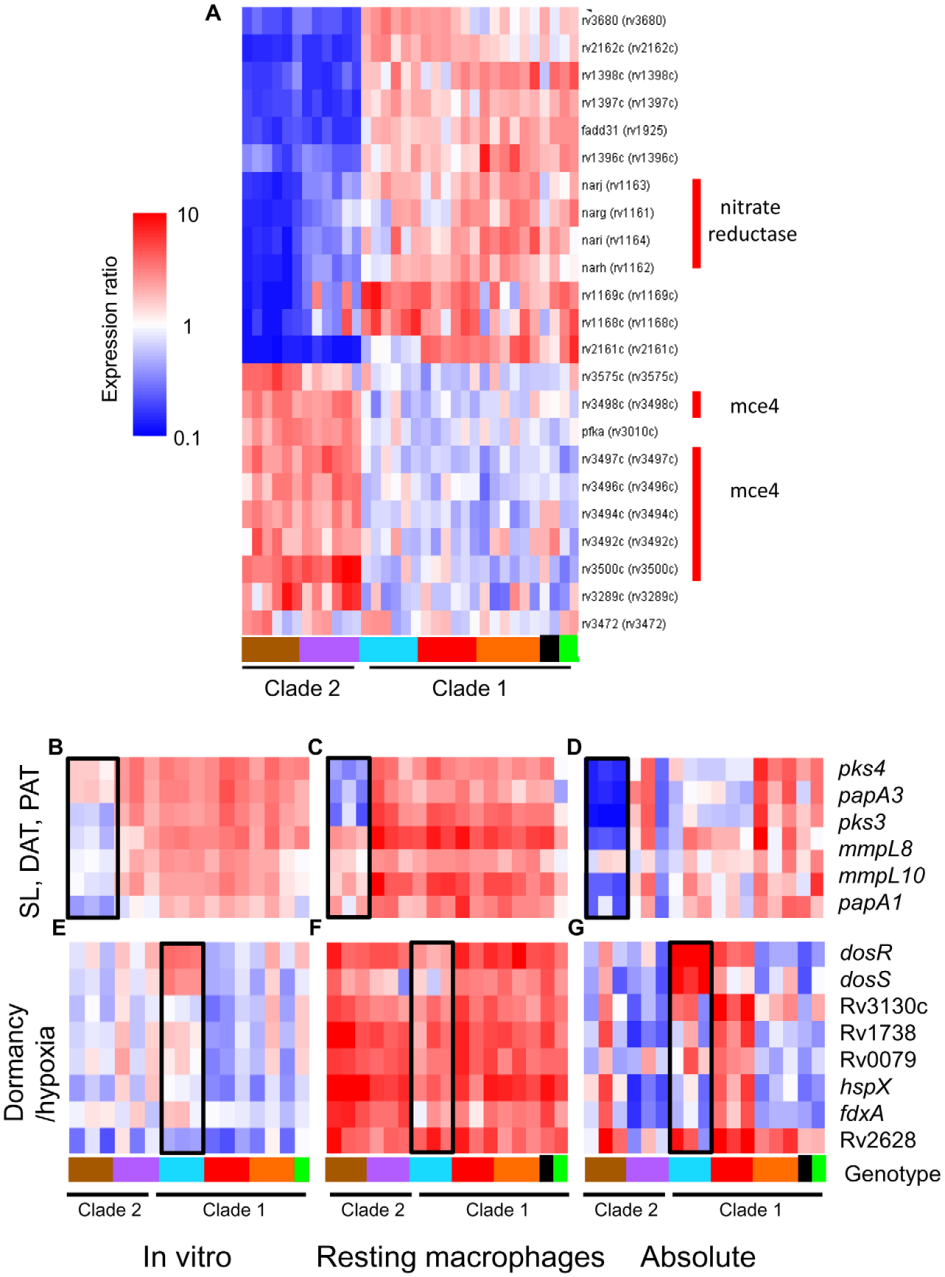
The additive effect of strain-specific differences in basal *in vitro* gene expression and transcriptional responses to intracellular cues. Microarray data from RNA derived from intracellular MTC 24h post-infection were compared directly following median normalization. This allows quantitative estimation of cumulative “absolute” intracellular transcript levels resulting from *in vitro* differences plus phagosome regulation. (A) Gene tree showing clade-specific intracellular expression levels of putative virulence factors. The *narGHJI* nitrate reductase operon, implicated in anaerobic nitrate respiration [84] and required for tissue-specific persistence in animal models [65], [85], is expressed at lower levels in all clade 2 versus clade 1 strains. The *mce4* operon, encoding a cholesterol import system required for growth in activated macrophages and persistence in mouse lungs [30], is overexpressed in clade 2 strains. (B–D) Reduced expression of sulfolipid (SL), diacyltrehalose (DAT), and polyacyltrehalose (PAT) synthesis genes in intracellular *M. africanum* West African 2 strains (B) due to lower *in vitro* expression (B) coupled with lower induction (*mmpL8*, *mmpL10*, *papA1*) or repression (*pks4*, *papA3*, *pks3*) in phagosomes of resting macrophage (C). (E–G) Expression and regulation of DosRS dormancy regulon in MTC clinical isolates. Despite *in vitro* overexpression of the *dosRS* two-component regulator in Beijing strains, transcripts of downstream effectors were not notably elevated (E). *dosRS* and effectors were induced in all strains by phagosomal cues in resting (F) and activated macrophages (data not shown). Although “absolute” levels of *dosRS* are highest in Beijing strains, DosR-dependent genes are expressed more highly in Haarlem strains (G). Black box in genotype legend denotes reference strain CDC1551. A wider scale for gene expression (0.1–10) was used for (A,D,G).

The success of mycobacteria/host cell interactions is likely impacted by both absolute transcript levels as well as the appropriate, temporal regulation of gene expression. In the previous section, we normalized intracellular array data to strain-matched extracellular controls to focus on relative responses to the phagosomal environment rather than inherent strain-dependent differences. To assess the additive effect of baseline *in vitro* differences and phagosomal changes in transcription, we directly compared transcript levels from intracellular MTC isolates. Although there are potential caveats this *in silico* analysis, such as skewing of values due to normalization of data from separate hybridizations, the constitutive expression of housekeeping genes (i.e. *sigA*) and qRT-PCR analyses discussed below (Supplementary Figure S10) served to validate this approach. This analysis yielded a robust differentiation of phylogenetic relationships which discriminated between strains, genotypes, and clades (Supplementary Figure S6). For example, the clade 2-specific underexpression of the nitrate reductase (*narGHJI*) and overexpression of the *mce4* locus (Rv3492c-3501c) (Figure 6A) present candidate mechanisms for the reduced fitness of these strains for intramacrophage survival (Figure 3). Closer examination of *narG* expression by qRT-PCR revealed that diminished *in vitro* expression coupled with lack of induction inside macrophages both contribute to >10-fold lower transcript levels in West African 2 strains within the phagosome (Supplementary Figure S10A). This analysis identified 499 and 463 genes from resting and activated macrophages, respectively, with significant (p<0.01) genotype-dependent differences in phagosomal transcript levels. Differences in the expression level of other genes involved in toxin-antitoxin pairs (Rv0549c-0550c), metabolism (*glgC*), DNA repair (*nrdF,nrdEIH*), and transcriptional regulation (*furB*, *whiB4*, Rv0452) point to additional mechanisms by which natural variation in gene expression may impact mycobacterial pathogenesis.

To further illustrate the integration of our multilevel array analysis of MTC clinical isolates, Figure 6B–G depicts a comprehensive examination of the *in vitro*, intracellular, and cumulative transcriptional profiles of two sets of genes implicated in mycobacterial pathogenesis. The polyketide synthesis loci *mmpL8-papA1-pks2* and *pks3-pks4-papA3-mmpL10* are required for the synthesis of sulfolipid (SL) and di- and poly-acyltrehaloses (DAT, PAT) respectively [33], [34], [35]. The *in vitro* underexpression of SL synthesis genes and the repression of DAT/PAT gene expression in macrophages in West African 2 strains results in dramatic cumulative differences in the transcript levels of these genes when the bacilli are inside phagosomes (Figure 6B–D, Supplementary Figure S10C). The DosR regulon, induced by the DosRS two-component regulator in response to hypoxia, carbon monoxide, and nitrosative stress (nitric oxide), is important for survival in animal disease models [36]. Despite the constitutive overexpression of *dosRS* by Beijing strains *in vitro* (Figure 6E), the *dosR* regulon was induced by phagosome cues in all strains (Figure 6F), with relative transcript levels of *dosR*-dependent effectors actually higher in several non-Beijing vs Beijing isolates (Figure 6G, Supplementary Figure S10D, E). Our findings argue that further characterization of MTC clinical isolates is warranted to understand the interplay between genotype, transcriptional regulation of gene expression, and virulence associated phenotypes.

## Discussion

The longstanding view of pathogenic mycobacteria as a homogeneous clonal population with negligible functional genetic diversity has been challenged by recent molecular genotyping studies [2] and whole genome sequencing of members of the MTC [37]. This new appreciation of the significant genetic diversity among MTC isolates has prompted studies documenting strain-dependent differences in resistance to *in vitro* stress [6], [7], [38], interactions with host cells [8], [26], [27], [39], [40], [41], and virulence in animal infection models [9], [10], [11], [12], [13], [42], [43], [44]. Phylogenetic analyses of the MTC have delineated multiple distinct lineages broadly divided into “modern” (clade 1) and “ancient” (clade 2) strains, with the latter including animal pathogens with distinct host tropisms [1], [2], [45], [46]. Although molecular genotyping provides a robust framework for understanding evolution and epidemiology of MTC, the functional impact of genetic diversity remains poorly characterized. Our microarray data revealed phylogenetically-constrained patterns of *in vitro* gene expression including clade-, genotype-, and strain-specific signatures. The dramatic effect of natural genetic diversity on transcriptome profiles, brought about by gene deletion, promoter SNPs, or missense mutations in regulators or other proteins, has yielded a functionally heterogeneous population of pathogenic mycobacteria.

The constitutive dysregulation of loci known to impact mycobacterial pathogenesis highlights putative mechanisms underlying the spectrum of host interactions and disease outcomes caused by MTC clinical isolates. For example, clade 2 isolates of the West African 2 and EAI genotypes showed elevated transcription of the *mce4* locus (Figure 6, Supplementary Figure S9A). The 11-gene *mce4* operon encodes a cholesterol import system that is dispensable for growth in resting macrophages during early infection but is essential for growth in interferon-γ activated macrophages in order to maintain persistent infections in the mouse model [30], [47], [48], [49], [50]. Given the repression of the cholesterol uptake genes by all strains upon macrophage infection, perhaps to counter the toxicity associated with utilization of cholesterol [24], the elevated basal expression of the *mce4* locus could conceivably contribute to the reduced fitness of clade 2 strains in macrophages. Altered expression of virulence regulators could also profoundly influence mycobacteria-host interactions. Clinical isolates of the EAI genotype constitutively overexpressed *virS* (Figure 2B, Supplementary Figure S8 and S9C), an AraC-family transcriptional regulator found only in the MTC [51]. VirS is reportedly required for the expression of the adjacent, divergently transcribed *mymA* operon (Rv3083-Rv3089) which acts to modify mycolic acid components of the cell wall during acid stress [52], [53]. Disruption of either *virS* or *mymA* results in altered cell wall composition and structure, increased sensitivity to antibiotics and surface-acting stresses, and attenuation of virulence in activated macrophages and guinea pigs [52], [53]. In contrast to Singh *et al.* [53], our data suggest that VirS represses rather than activates transcription of the *mymA* locus. The dramatic repression of the *mymA* operon in EAI strains overexpressing *virS* is likely to have deleterious effects on virulence-associated phenotypes.

Following establishment of the baseline transcriptomes of the clinical strains in rich medium, we characterized the transcriptional responses of the bacteria following infection of resting and activated macrophages in the context of their intracellular growth profiles. This analysis revealed both common and divergent responses that paralleled the capacity of MTC isolates to survive intracellularly, albeit with varying degrees of success. The common responses constitute a rigorous delineation of the core transcriptome associated with intracellular survival of *Mycobacteria*. Many of the “themes” revealed in the core transcriptome, consisting of 280 genes with 168 genes induced and 112 genes repressed in the intracellular environment, validate the findings reported in earlier studies [22], [23]. The ubiquitous expression and conserved regulation of these genes within macrophages across a global panel of strains should inform the prioritization of targets for the development of novel biomarkers, drugs, or vaccines. This data also suggest that the 45% (75/168) of the core up-regulated genes with no known function, many of which are unique to *Mycobacteria*, warrant further investigation.

In contrast to the common responses, the distinct intramacrophage fitness levels and intra-strain diversity of transcription profiles, especially between clade 1 and clade 2 strains, were clear indicators of unique lineage-specific adaptation to the phagosome environment. It is notable that gene expression differences were evident as early as 24hr post-infection before growth/survival of strains diverged significantly. The identification of lineage-specific regulons (both known virulence factors and genes of unknown function) that correlate with phenotypes relevant to pathogenesis (i.e. survival in macrophages) provides a powerful approach for discovery of novel strategies of host adaptation and virulence. Differential regulation of virulence factors by *M. africanum*, the most phylogenetically distant genotype in this study, may provide insight into the evolution of Mtb-host interactions. Notably, the West African 2 strains were distinguished by their failure to upregulate genes required to synthesize sulfolipids (SL-1, *pks2-papA1-mmpL8*) and phthiocerol dimycocerosates (PDIM, *ppsABCDE,drrABC*) within macrophage phagosomes (Supplementary Figure S10B, S10C). The importance of SL-1 and PDIM during infection [54], [55], [56] stem from multiple roles including cell well integrity, modulation of the immune response, resistance of interferon-γ independent defenses, and detoxification of proprionate and maintaining redox balance during lipid metabolism [55], [56], [57], [58], [59]. Similarly, high levels of hypoxia-inducible nitrate reductase activity, which allows nitrate respiration in the absence of oxygen, are associated with the globally dominant lineages of the MTC [60], [61], [62]. West African 2 isolates, however, expressed the *narGHJI* nitrate reductase at depressed levels *in vitro* and failed to induce both *narGHJI* and the nitrate/nitrite transporter *narK2*X inside phagosomes (Supplementary Figure S10A). This is consistent with clade 2-specific SNPs in the promoters of both *narGHJI* and *narK2X* [61], [63], [64]. Although animal studies suggest nitrate reductase is required for virulence [65], the exact role of this virulence factor is unclear given that nitrate reductase does not appear to support actual anaerobic growth [66]. Thus, evolving the ability to respond to host-derived and endogenous metabolic cues and adapt to conditions within the host represent key steps in the continuing evolution of the MTC.

On the other hand, the Beijing genotype represents a modern lineage whose growing predominance in the clinical setting suggests a selective advantage over other *M. tuberculosis* strains. Speculated mechanisms underlying the success of Beijing isolates have included escape from BCG vaccination, enhanced multidrug resistance, and expression of unique virulence lipids [67]. A recent study by Reed *et al.* found that a large subset of Beijing strains constitutively overexpress the *dosRS* two-component regulator and 48-gene regulon [20]. The resulting induction of a triacylglycerol (TAG) synthase (Rv3130c) and accumulation of fatty acids in the form of TAG was hypothesized to augment virulence by affording a selective advantage during intracellular survival or latency. As shown in Figure 3, the survival and intracellular replication of Beijing isolates within murine macrophages was unremarkable relative to other lineages, regardless of macrophage activation state. Although our data confirmed the elevated basal transcription levels of *dosRS* in Beijing isolates (Figure 2B and 6F, Supplementary Figures S8 and S9B), transcript levels of *dosRS*-dependent effectors such as *hspX* and Rv3130c were comparable to reference strains (CDC1551 and H37Rv). The use of microaerophilic static growth conditions in this study, leading to moderate activation of the DosR regulon and accumulation of TAG (data not shown) compared to aerated growth in roller-bottles would explain the discrepancy with the findings of Reed *et al.* [20], [68]. During intracellular adaptation, the induction and overall expression of *dosRS* and downstream genes was lower in Beijing strains likely as a consequence of genotype-specific frameshift mutations in *dosT* [68], encoding a histidine kinase that acts on DosR [69], [70]. Our findings do not support the hypothesis that overexpression of *dosRS* by Beijing strains provides a selective advantage for survival within macrophages. The molecular mechanisms behind the success of this lineage remain to be elucidated.

The global predominance of strains belonging to “modern” clade 1 compared to “ancestral” clade 2 lineages would support the hypothesis that transcriptome features associated with these strains represent strategies evolved for enhanced human infection and transmission. Recent evidence suggests that a strong and stable association exists between MTC strains and distinct geographic regions and human populations [71], [72], [73]. Thus, selective pressures unique to their “native” host environment may be driving the genetic divergence of MTC strains.

Finally, the global resurgence of tuberculosis necessitates a concerted effort to improve the outdated and inadequate strategies of prevention, detection, and treatment that are currently available. Characterization of the molecular and phenotypic differences and similarities between clinical isolates targeted by such interventions is a crucial component in the design of new control measures. In fact, mathematical modeling analyses by Cohen *et al.* [74] predicted that a failure to consider mycobacterial strain diversity could have a significant negative impact on vaccine efficacy, due to strain replacement by MTC variants not targeted by the vaccine. The functional genetic diversity and distinct macrophage interactions evident in our panel of strains reinforces the need to include challenges with diverse MTC strains as a routine step in vaccine testing [74] and drug screening.

## Materials and Methods

### Bacterial and cell culture

Fifteen well-characterized clinical isolates of the *M. tuberculosis* complex (MTC) representing five different phylogenetic genotypes, were selected based on previous studies on the genetic diversity of the MTC. All strains were characterized by various genotyping methods and susceptibility testing as described elsewhere (Supplementary Figure S1, Supplemental Table S1) [2]. Two fully sequenced reference *M. tuberculosis* strains, H37Rv and CDC1551, and all clinical isolates were routinely cultured in 7H9-OADC medium in 25-cm^2^ vented flasks without shaking. The MTC clinical isolates used in this study were handled to minimize *in vitro* passaging. Briefly, strains were initially cultured from clinical samples on Lowenstein-Jensen (LJ) medium for routine diagnostic testing at the National Reference Center in Borstel, Germany. Clinical isolate cultures used in this study were derived from frozen stocks prepared after a single *in vitro* passage of original archived samples. The clinical isolate reference strain CDC1551 was also maintained to minimize serial passaging. The virulent lab strain H37Rv, however, has undergone countless rounds of *in vitro* passaging. For *in vitro* growth assays, the OD_600_ (BioRad Smart Spec 3000) of 15ml cultures inoculated at OD_600_ ∼0.05 from frozen stocks grown to mid-log phase was monitored. Bone marrow-derived macrophages (MØ) were isolated from C57BL/6 mice and grown in DMEM supplemented with 10% FCS, 1% sodium pyruvate, 1% L- glutamine, 20% L cell-conditioned medium and 1% penicillin and streptomycin. Media lacking antibiotics was added at least 24h before initiation of mycobacterial infections and maintained during infection.

### Macrophage survival assays

To monitor the survival/growth of MTC strains in macrophages, confluent macrophage monolayers (∼5×10^5^ cells) in 24-well dishes were infected at an MOI ∼1∶1. At 0, 2, 4, 7, and 11 days post-infection, intracellular MTC were released from monolayers by lysis with ddH_2_O+0.05% Tween-80, serially diluted in PBS+0.05% Tween-80, and plated on 7H10 + cycloheximide (10mg/ml) agar. Colony forming units were enumerated after ∼3–4 weeks incubation at 37°C. Activated macrophages were primed with 100 units/ml of recombinant mouse interferon-γ (Peprotech) for 24h followed by pre-activation with 10ng/ml of LPS (Sigma) for 16hr. The integrity of the monolayer was confirmed at each time point by visual microscopic inspection. A total of 6 wells/strain were analyzed (two independent biological replicates with three wells/strain in each assay).

### Macrophage infections and RNA isolation

The protocol for RNA isolation from intracellular MTC has been previously described [22]. Briefly, confluent monolayers of ∼2×10^7^ MØ in 75-cm^2^ vented tissue culture flasks were infected (MOI ∼4∶1) with MTC strains grown to mid-log phase (OD = 0.6–0.8) in standing 75-cm^2^ vented flasks. Control samples consisted of an aliquot of the same bacterial preparation used to infect macrophages that was instead placed in a 75- cm^2^ flask with no macrophages. Care was taken to treat control bacteria identically with the exception of the condition being tested to minimize detection of spurious changes in gene expression due to irrelevant stimuli. Prior to infections, bacteria were declumped by 10 passages through a 21 gauge needle in PBS + 0.01% Tween-80 and diluted in 1ml of uptake buffer (PBS with 4.5mg/ml glucose, 5mg/ml defatted BSA, and 1mg/ml gelatin) and 5ml binding medium (DMEM with 5% fetal calf serum, 10mM HEPES, pH 7.4). After 4h infection, extracellular bacteria were removed and replaced with fresh macrophage medium without antibiotics. At 24h post-infection, addition of GTC lysis buffer (4M guanidine thiocyanate, 0.5% Na N-lauryl sarcosine, 25mM sodium citrate, and 0.1M β-mercaptoethanol) selectively lysed MØ, halted RNA transcription/degradation while leaving mycobacteria intact as previously described [75], [76]. Extracellular controls remained in macrophage medium for the same 24h time course before being stopped by the addition of GTC lysis buffer. RNA from intracellular MTC strains and extracellular controls was isolated and linearly amplified (using 250ng total RNA template per reaction) as described previously [22]. Intracellular transcriptome profiling was conducted on two independent biological replicates for each strain in each macrophage type.

For *in vitro* array analyses, log-phase mycobacteria in starter cultures grown to mid-log from frozen stocks were inoculated into 7H9-OADC medium in 25-cm^2^ vented flasks at an OD ∼0.05 and grown without shaking for ∼1 week to an OD∼0.5–0.6. As described above, GTC lysis buffer added at a 5∶1 ratio to culture aliquots served to preserve RNA transcripts for RNA isolation and amplification. The results presented represent the average of three independent biological replicates.

### Target preparation and microarray hybridization

Amino-allyl modified amplified RNA (aRNA) were labeled with Alexa Fluor 555 (extracellular controls) and Alexa Fluor 647 (intracellular) (Invitrogen) and purified using a MegaClear kit (Ambion). 10µg of Alexa-labeled aRNA from paired samples was dried using a Speedvac and resuspended in 75µl of hybridization buffer (5× SSC, 25% formamide, 0.1% SDS, and 25µg salmon sperm DNA). Samples were denatured at 95°C for 5min and briefly cooled to 60°C before being applied to arrays under a glass LifterSlip (Erie Scientific). Slides were prehybridized for 1h in 25% formamide, 5× SSC, 0.1% SDS, 1% BSA and washed with H_2_O and isopropanol. Labeled targets were hybridized to microarrays in humidified slide chambers (Corning) at 45°C for 16–18 h. Arrays were washed sequentially with buffer 1 (2× SSC, 0.1% SDS) pre-warmed to 45°C, buffer 2 (0.2× SSC, 0.1% SDS), buffer 3 (0.2× SSC), and buffer 4 (0.05× SSC). Slides were briefly dipped in de-ionized ultrafiltered water before being dried by centrifugation (700×g, 10min).

For *in vitro* arrays, aRNA from clinical isolates was labeled with Alexa 647 while aRNA from log-phase strain CDC1551 labeled with Alexa 555 served as a common denominator on all slides. For macrophage infection arrays, the intracellular transcriptome of each strain was compared to a matched sample of the same strain from a macrophage-free control to measure relative changes in gene expression in response to the phagosome environment.

### Microarray fabrication

The microarray platform used can be accessed via NCBI's Gene Expression Omnibu (GEO) database [77] under platform accession number GPL5754.

### Microarray data analysis

Microarrays were scanned with a GenePix 4000B instrument (Axon Instruments, Inc.) with preliminary image analysis, spot intensity determination, background measurements, spot quality assessment and flagging conducted using Imagene software (version 6.0, Biodiscovery). Poor quality spots with signal intensities less than three standard deviations above background were excluded from further analysis. Subsequent normalization, statistical analysis, and visualization of array data were performed with Genespring 7.3 (Agilent). Genes with significant changes in expression levels relative to controls were identified based on fold change (1.5-fold) and reproducibility between replicates (p-value <0.05). One-way ANOVA and Tukey post-hoc tests were used to identify genes with significant differences in expression between conditions. For most analyses, multiple testing corrections (Benjamini and Hochberg false discovery rate <0.01) were applied. For the direct comparison of intracellular transcript levels (see Figure 6), instead of comparing with cohybridized extracellular control samples, preprocessed images from the channel representing intracellular samples were loaded into Genespring as a single-color experiment. Per chip (normalized to 50^th^ percentile) and per gene (normalized to median) normalizations were conducted on the assembled datasets. The same approach was employed to directly compare transcriptomes of strains from extracellular macrophage-free controls. A high correlation between the relative transcript levels of constitutive housekeeping genes (i.e. *sigA*) across the panel of clinical isolates analyzed in this manner served to validate this approach. The relative abundance of select transcripts expressed by intracellular MTC was further confirmed by qRT-PCR (Supplementary Figure S10).

All microarray data has been deposited in the GEO database under the accession number GSE21114 and is also available through the TBDB database at www.tbdb.org [78].

### Quantitative real-time RT-PCR (qRT-PCR)

RNA amplification and microarray methodology used herein were previously validated by qRT-PCR of select genes differentially regulated by the reference strain CDC1551 during infection of bone marrow derived macrophages [22]. Additional qRT-PCR validation of target gene expression of clinical isolates in this study was conducted by two-step real-time RT-PCR using iScript and iTaq SYBR Green reagents (Biorad). Each sample was analyzed in triplicate on an ABI 7500 starting with 100ng of total RNA (amplified and unamplified). C_T_ values were normalized to values obtained for *sigA*, a constitutively expressed Mtb gene, and relative changes in gene expression were calculated using the 2^−ΔΔCT^ method [79].

The faithful representation of transcript levels following linear amplification was further validated in this study by qRT-PCR of select genes by comparing expression ratios derived from unamplified versus amplified RNA templates prepared from clinical isolates (Supplementary Figure S8). For qRT-PCR analysis of *in vitro* and intracellular gene expression, aRNA from a single representative strain from each genotype served as template – Haarlem (4130/02), Beijing (12594/02), Uganda (2333/99), EAI (4850/03), and West African 2 (10514/01).

### Statistical analysis

Preliminary analysis of *in vitro* growth data and intramacrophage survival/growth data was performed using GraphPad Prism software (version 4). Statistical analysis of growth data shown in Figure 3 and Supplemental Figure S2 were conducted using JMP 8.0 software (SAS) with assistance provided by the Cornell Statistical Consulting Unit (CSCU). Significant differences (p<0.05) were determined by one-way ANOVA with pair-wise comparisons between strains made using the Tukey-Kramer HSD post-hoc test.

## Supporting Information

Figure S1 Molecular genotyping analysis of MTC clinical isolates in this study, including 24-loci MIRU-VNTR (Mycobacterial Intespersed Repetitive Units-Variable Number of Tandem Repeats), *IS6110* RFLP (Restriction Fragment Length Polymorphism), and spoligotyping (spacer oligonucleotide typing). Note the distinction of clade 1 strains (including Uganda, Beijing, and Haarlem genotypes and reference strains H37Rv and CDC1551) from clade 2 strains (EAI and West African 2 genotypes). More complete description of mycobacterial strains can be obtained from http://www.miru-vntrplus.org/.
(0.40 MB TIF)

Figure S2 *In vitro* growth kinetics of MTC clinical isolates. The growth of mycobacterial strains was monitored for 20 days by optical density (OD_600_) measurements of 15ml standing cultures in 7H9 liquid medium. Growth of *M. tuberculosis* Haarlem (A), Uganda (B), Beijing (C), EAI (D), and *M. africanum* West African 2 (E) strains are shown relative to reference strains H37Rv and CDC1551 (green lines). Colors for each genotype correspond to colors used in Fig. 1A phylogenetic tree and throughout the manuscript.
(3.52 MB TIF)

Figure S3 Condition tree of genes with clade-specific expression profiles *in vitro*. One-way ANOVA (using Benjamini and Hochberg False Discovery Rate p<0.01) of *in vitro* transcriptome data from log phase bacteria identified 156 genes with significant differences in basal expression levels between clade 1 and clade 2 strains. The condition tree (Pearson correlation) generated from this gene list clearly delineates strains at the clade and genotype level based on differential transcription patterns (indicated by coloring of branches and key shown below the tree). Expression data for individual genes was clustered (vertical order) using the distance measure.
(0.59 MB TIF)

Figure S4 Genes with strain specific *in vitro* transcription patterns. Raw data were derived from array analysis of 16 MTC strains relative to CDC1551 reference strain in log phase growth in 7H9 medium (3 biological replicates each). One-way ANOVA of a quality-filtered gene list (genes flagged present in 42 of 48 samples) using Benjamini and Hochberg False Discovery Rate p<0.01 identified 195 genes with strain-specific expression. The matrix shows the results of pair-wise comparisons between strains using the Tukey post hoc test. The numbers within red squares indicate genes with unique expression patterns between the two intersecting genotypes.
(1.71 MB TIF)

Figure S5 Genes displaying conserved induction (A) or repression (B) in both resting and activated macrophage phagosomes (24h post-infection) across our panel of MTC clinical isolates. Raw data were derived from array analysis of 17 MTC strains comparing intracellular transcript levels to extracellular controls of the same strain. “Universal” genes were selected as detailed in Figure 4 legend, with a subset of genes shown here. Genotypes are indicated by the color bar at bottom, which corresponds to color code shown in Figure 1A. Black indicated CDC1551 and green indicates H37Rv. Samples from both resting (white) and activated (gray) macrophage were included. (A) Universally induced genes included members of the DosR dormancy regulon (*Rv2032*, *Rv2626c*, *Rv3130c*, *Rv1738*, *hspX*, *Rv3133c*, *Rv2028c*), pH stimulon (*lipF*), ROI stress response (*furA*, *fdxD*, *Rv1460-1461*, *fdxA*), general stress (*dnaJ*, *hspR*, *dnaK*, *grpE*, *groEL2*, *hsp*), regulation (*whiB7*, *furA*, *sigB*, *whiB6* (Rv3862c)), and lipid metabolism (*mez*, *icl*, *fadD9*). (B) Universally repressed genes included Type I NADH dehydrogenase (*nuo*), ATP synthase (*atp*), mycolic acid synthesis (*acpM*, *fabD*, *kasA*, transcriptional regulators (i.e. *cspA*, *sigD*, Rv3676), putative lipid transporters *mce1* (Rv0167, Rv0171, Rv0172), *mce4* (Rv3492c, Rv3493c, Rv3497c), *mmpL9*, *mmpS2*, *mmpL12*, and numerous lipoproteins (*lppL*, *lpqM*, *lprO*, *lprJ*, *lprE*, *lprL*, *lprK*).
(1.96 MB TIF)

Figure S6 Direct comparison of transcript levels from intracellular MTC isolates after 24h infection of resting macrophages. Microarray images were loaded into Genespring as a single color experiment and normalized against the median. This effectively assesses the additive effect of baseline *in vitro* differences and intracellular changes in gene expression. Condition tree (Spearman correlation) of clinical isolates based on 499 genes determined by one-way ANOVA to exhibit genotype-dependent profiles (using Benjamini and Hochberg False Discovery Rate p<0.01). Compare with phylogenetic tree (Fig. 1A) and note clustering of strains according to genotype and delineation of clade 1 and clade 2 strains based on “absolute” intracellular gene expression.
(0.80 MB TIF)

Figure S7 MTC clinical isolates display significantly different growth and survival profiles *in vitro* and in murine macrophages. The results of ANOVA and pair-wise comparisons between strains using the Tukey-Kramer HSD are summarized in connecting letter reports. Strains that do not share a letter (A–G in growth profile column) are significantly different (p<0.05). In A), for example, 2169/99 is significantly different from all other strains whereas 4130/02 is similar to 2336/02 and 2333/99. Strains are ranked from top to bottom by least-squares differences means which correlates with the growth/fitness of strains *in vitro* (A), in resting macrophages (B), or activated macrophages (C). Strain names are color coded to indicate genotype (red = Haarlem, blue = Beijing, orange = Uganda, purple = EAI, brown = West African 2). Clinical isolates belonging to clade 1 are shown with a white background while clade 2 isolates are highlighted in gray.
(1.88 MB TIF)

Figure S8 Validation of linear RNA amplification by qRT-PCR. Unamplified and amplified RNA from log phase *in vitro* mycobacteria including CDC1551 (reference strain), Beijing strain 12594/02, and EAI strain 4850/03 was reverse transcribed and quantified by relative qRT-PCR by normalization to *sigA*. Expression ratios shown are relative to the reference strain CDC1551. Solid bars and open bars represent the use of unamplified and amplified RNA, respectively. There was an excellent correlation between expression ratios derived from amplified and unamplified RNA. Note the overexpression of the *dosR* two-component response regulator in the Beijing strain. Although the *dosR*-dependent gene Rv3130c is expressed higher in Beijing versus EAI, transcript levels are comparable between 12594/02 and the reference strain, CDC1551. The overexpression of *virS* and concomitant repression of Rv3083 in EAI versus Beijing and CDC1551 verifies the genotype-specific regulation of this operon. Error bars indicate the standard deviation of ΔΔC_T_ values calculated as described in the Guide to Performing Relative Quantitation of Gene Expression Using Real-Time Quantitative PCR (ABI).
(0.35 MB TIF)

Figure S9 qRT-PCR analysis of select mycobacterial virulence factors exhibiting strain-dependent expression profiles *in vitro*. A) Overexpression of *mce4C* (involved in cholesterol uptake and metabolism) in West African 2 and EAI (clade 2 strains). B) Beijing genotype specific overexpression of *dosR* (response regulator of the hypoxia/dormancy regulon) but not *dosR*-dependent effectors (*hspX*, Rv3130c). C) EAI genotype specific overexpression of *virS* transcriptional regulator and repression of *virS*-dependent Rv3083. The color legend shown in A), which corresponds to colors used in all figures to designate specific genotypes, was also applied in B) and C). See legend for Supplementary Figure S8 for description of error bars.
(0.70 MB TIF)

Figure S10 qRT-PCR analysis of select mycobacterial virulence factors exhibiting strain-dependent expression profiles during intracellular growth within macrophages. Data labeled “Outside” at the left of each panel represents the direct comparison (as detailed in Materials and Methods) of transcript levels in macrophage-controls incubated in DMEM infection medium for 24hr at 37C. Similarly, data labeled “Inside” at the right of each panel reflects direct comparison of transcript levels from intracellular MTC 24hr post-infection. For each gene, expression ratios “outside” and “inside” are expressed relative to West African 2 following normalization to *sigA* from the same sample. “Macrophage” expression ratios represent changes of gene expression upon macrophage invasion relative to levels in extracellular, macrophage-free controls. A) The underexpression of *narG in vitro* (outside) and lack of intracellular induction (macrophage) in West African 2 strains leads to large differences in transcript levels within the phagosome (inside). B) Differential basal expression and intracellular induction of *ppsA* in West African 2 strains. C) Elevated expression of *dosR* by extracellular control Beijing strains coupled with dampened induction upon macrophage infection results in comparable levels of *dosR* in the phagosome across all genotypes. D) Intracellular transcript levels of the *dosR*-dependent triacylglycerol synthase Rv3130c were lowest in the Beijing genotype, reflecting the ∼10-fold lower induction inside macrophages. E) Reduced transcript levels of *pks2*, a polyketide synthase involved in synthesis of the cell wall component sulfolipid [32], [34], by West African 2 strains is a combined result of lower baseline expression *in vitro* and defective upregulation within the phagosome. See legend for Supplementary Figure S8 for description of error bars.
(1.03 MB TIF)

Table S1 Clade specific gene expression *in vitro* (93 genes, Clade 1>Clade 2). This list was based on one-way ANOVA analysis of *in vitro* transcriptome data from log phase bacteria (using Benjamini and Hochberg False Discovery Rate p<0.01). The values indicate expression ratios relative to the CDC1551 reference strain.
(0.05 MB XLS)

Table S2 Clade specific gene expression *in vitro*, (63 genes, Clade 2>Clade 1). This list was based on one-way ANOVA analysis of *in vitro* transcriptome data from log phase bacteria (using Benjamini and Hochberg False Discovery Rate p<0.01). The values indicate expression ratios relative to the CDC1551 reference strain.
(0.04 MB XLS)

Table S3 Universally induced genes in resting and activated macrophages. Identification of genes with conserved expression and regulation inside macrophage phagosome across global panel of MTC clinical isolates. Global gene expression profiles of 17 MTC strains 24h post-infection of resting and activated macrophages were determined by microarray. Normalized expression ratios for each strain were determined by comparison of RNA from intracellular bacteria versus control bacteria of the same strain treated identically except for phagocytosis by macrophages. “Universal genes” were selected based on trending up or down in all strains (up >1.2-fold in 15 of 17 strains) and significantly being induced in >50% of strains (up >1.5× in 8 of 17 strains) in each macrophage type. The starting gene list for this analysis included only genes flagged as present in the majority of samples from both macrophage types. Shown are the 168 genes universally induced in resting and activated macrophages (Figure 4a, white).
(0.13 MB XLS)

Table S4 Universally induced genes in resting macrophages only. See legend for Table S3 for details of analysis parameters. Shown are 57 genes induced by panel of MTC clinical isolates in resting macrophages only (Figure 4a, red).
(0.06 MB XLS)

Table S5 Universally induced genes in activated macrophages only. See legend for Table S3 for details of analysis parameters. Shown are 119 genes induced by panel of MTC clinical isolates in activated macrophages only (Figure 4a, blue).
(0.10 MB XLS)

Table S6 Universally repressed genes in resting and activated macrophages. See legend for Table S3 for details of analysis parameters. Shown are the 112 genes repressed in panel of MTC clinical isolates in resting and activated macrophages (Figure 4b, white).
(0.10 MB XLS)

Table S7 Universally repressed genes in resting macrophages only. See legend for Table S3 for details of analysis parameters. Shown are the 42 genes repressed in panel of MTC clinical isolates in resting and activated macrophages (Figure 4b, red).
(0.05 MB XLS)

Table S8 Universally repressed genes in activated macrophages only. See legend for Suppl. Table S4 for details of analysis parameters. Shown are the 152 genes repressed in panel of MTC clinical isolates in activated macrophages only (Figure 4b, blue).
(0.12 MB XLS)

Table S9 Activation-dependent genes (activated>resting) identified by one-way ANOVA of all intracellular transcription profiles (Benjamini and Hochberg False Discovery Rate p<0.01). Global gene expression profiles of 17 MTC strains 24h post-infection of resting and activated macrophages were determined by microarray. Normalized (lowess) expression ratios for each strain were determined by comparison of RNA from intracellular bacteria versus control bacteria of the same strain treated identically except for phagocytosis by macrophages.
(0.23 MB XLS)

Table S10 Activation-dependent genes (resting>activated) identified by one-way ANOVA of all intracellular transcription profiles (Benjamini and Hochberg False Discovery Rate p<0.01). See legend for Suppl Table S9 for explanation of expression values.
(0.29 MB XLS)
