# Supplementary figures and images for: Functional Genetic Diversity among Mycobacterium tuberculosis Complex Clinical Isolates: Delineation of Conserved Core and Lineage-Specific Transcriptomes during Intracellular Survival
Source: PLoS Pathog. 2010 Jul 8;6(7):e1000988. doi: 10.1371/journal.ppat.1000988 (PMC2900310; doi:10.1371/journal.ppat.1000988)

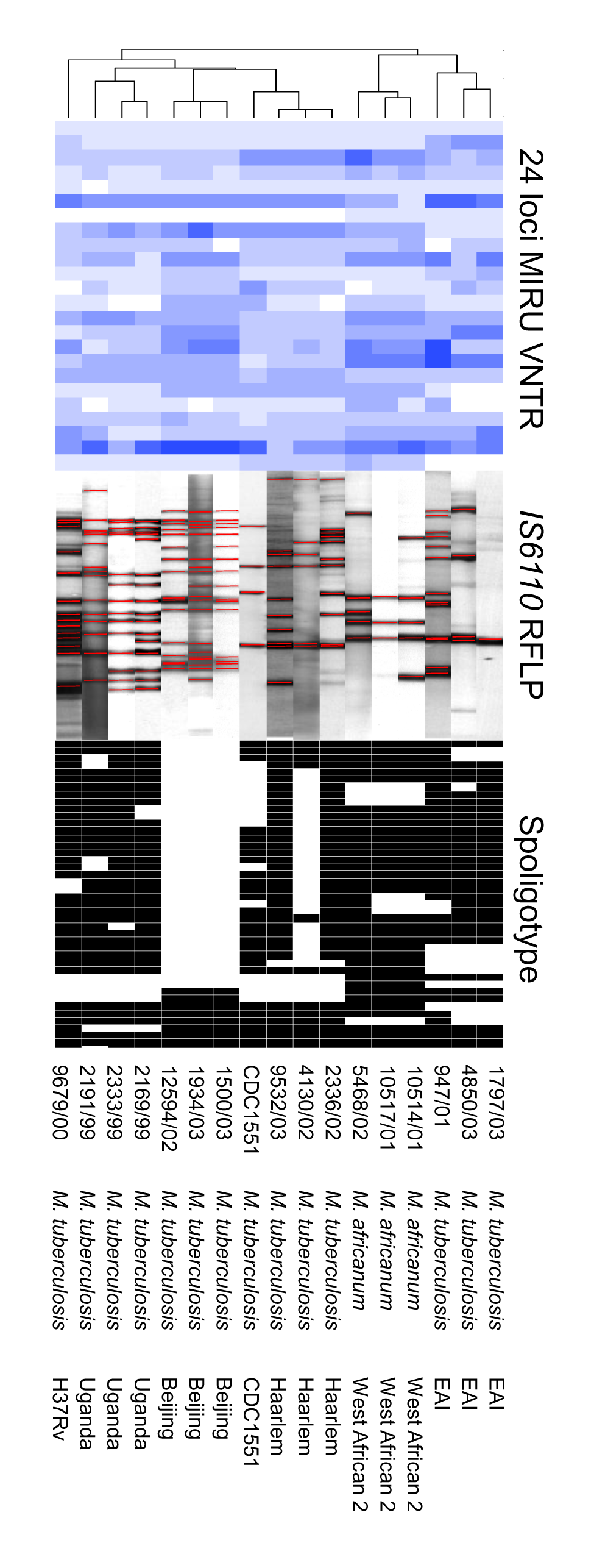

Supplement: Figure S1 — Molecular genotyping analysis of MTC clinical isolates in this study, including 24-loci MIRU-VNTR (Mycobacterial Intespersed Repetitive Units-Variable Number of Tandem Repeats), IS6110 RFLP (Restriction Fragment Length Polymorphism), and spoligotyping (spacer oligonucleotide typing). Note the distinction of clade 1 strains (including Uganda, Beijing, and Haarlem genotypes and reference strains H37Rv and CDC1551) from clade 2 strains (EAI and West African 2 genotypes). More complete description of mycobacterial strains can be obtained from http://www.miru-vntrplus.org/. (0.40 MB TIF) [file ppat.1000988.s001.tif]

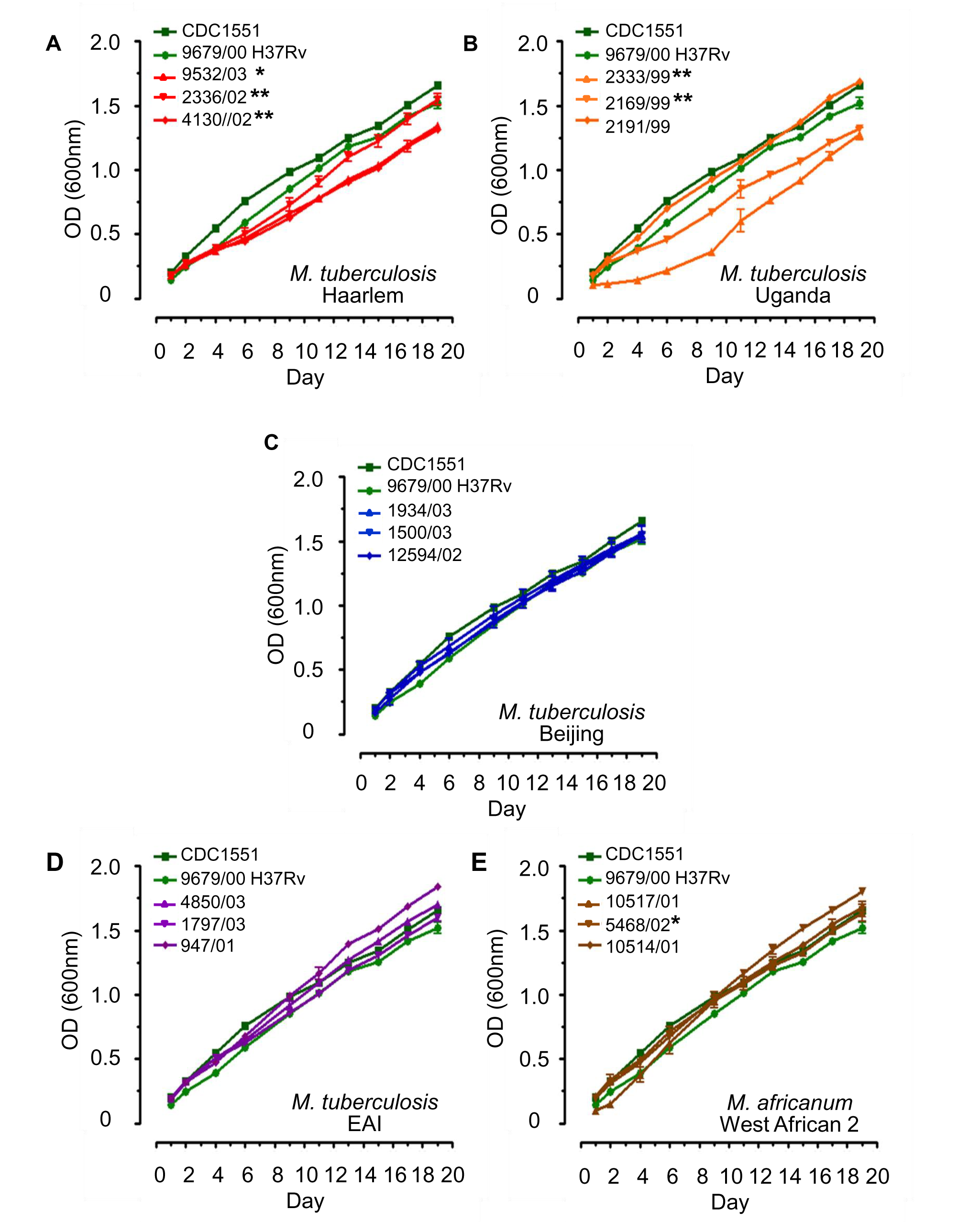

Supplement: Figure S2 — In vitro growth kinetics of MTC clinical isolates. The growth of mycobacterial strains was monitored for 20 days by optical density (OD600) measurements of 15ml standing cultures in 7H9 liquid medium. Growth of M. tuberculosis Haarlem (A), Uganda (B), Beijing (C), EAI (D), and M. africanum West African 2 (E) strains are shown relative to reference strains H37Rv and CDC1551 (green lines). Colors for each genotype correspond to colors used in Fig. 1A phylogenetic tree and throughout the manuscript. (3.52 MB TIF) [file ppat.1000988.s002.tif]

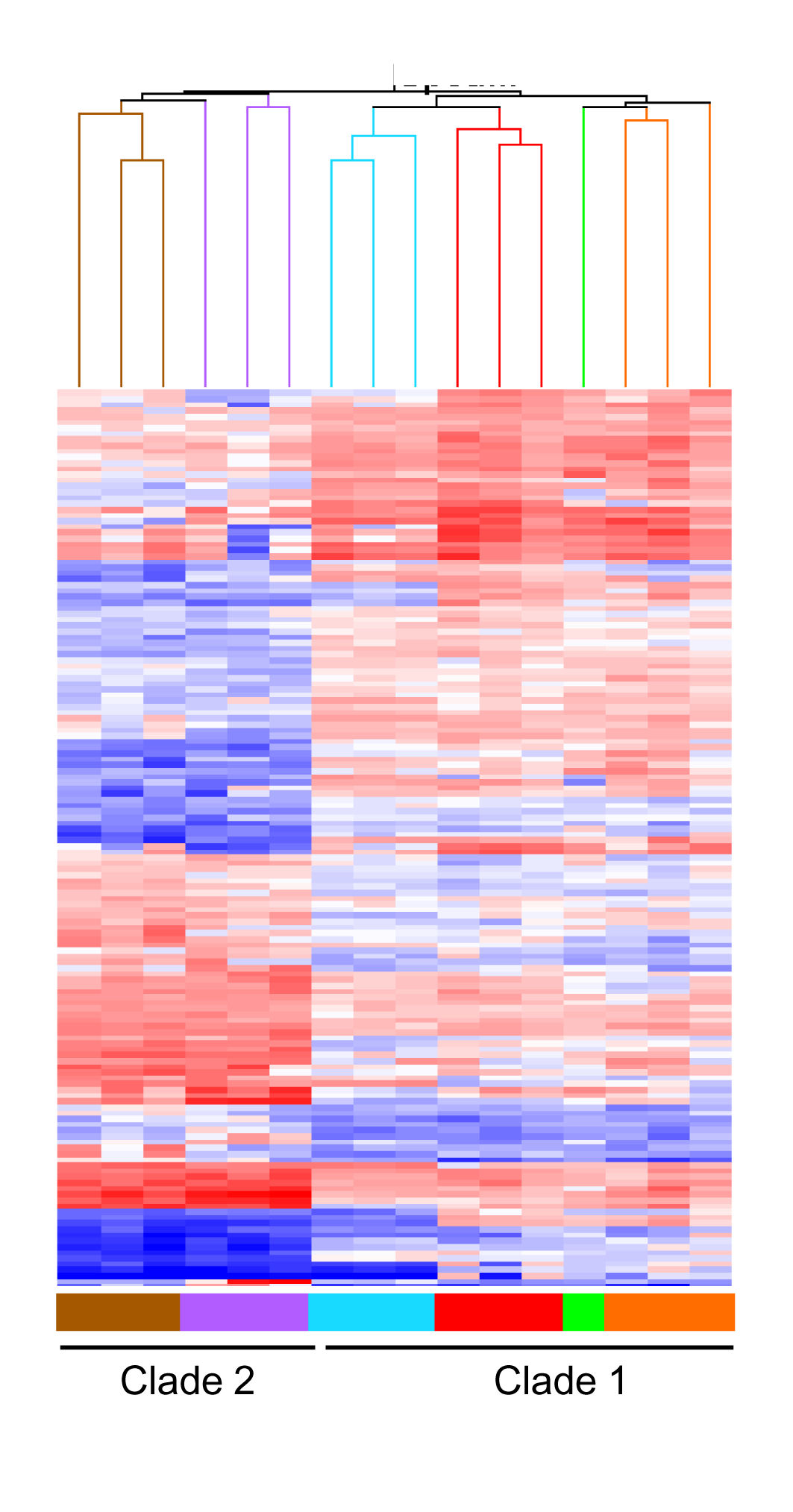

Supplement: Figure S3 — Condition tree of genes with clade-specific expression profiles in vitro. One-way ANOVA (using Benjamini and Hochberg False Discovery Rate p<0.01) of in vitro transcriptome data from log phase bacteria identified 156 genes with significant differences in basal expression levels between clade 1 and clade 2 strains. The condition tree (Pearson correlation) generated from this gene list clearly delineates strains at the clade and genotype level based on differential transcription patterns (indicated by coloring of branches and key shown below the tree). Expression data for individual genes was clustered (vertical order) using the distance measure. (0.59 MB TIF) [file ppat.1000988.s003.tif]

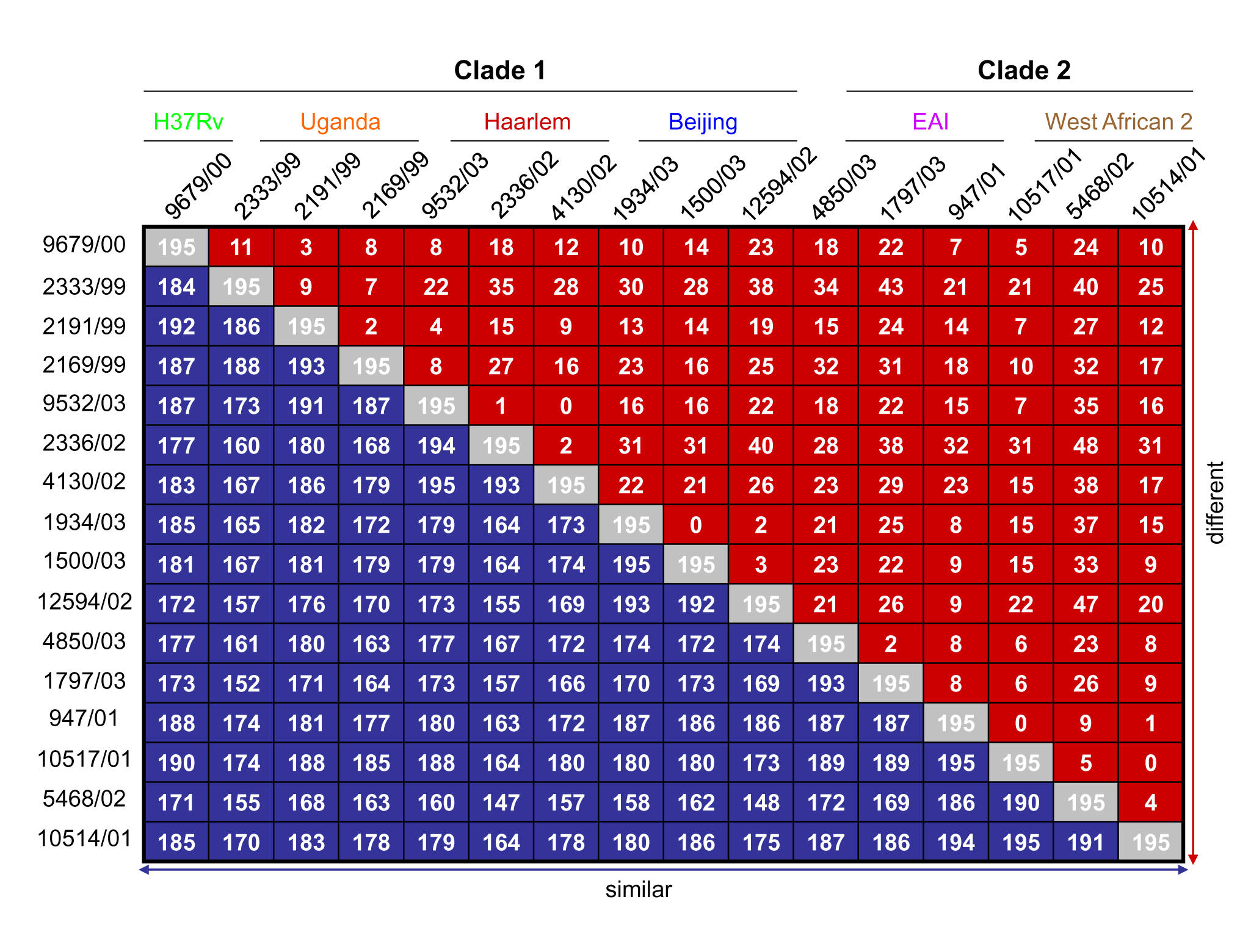

Supplement: Figure S4 — Genes with strain specific in vitro transcription patterns. Raw data were derived from array analysis of 16 MTC strains relative to CDC1551 reference strain in log phase growth in 7H9 medium (3 biological replicates each). One-way ANOVA of a quality-filtered gene list (genes flagged present in 42 of 48 samples) using Benjamini and Hochberg False Discovery Rate p<0.01 identified 195 genes with strain-specific expression. The matrix shows the results of pair-wise comparisons between strains using the Tukey post hoc test. The numbers within red squares indicate genes with unique expression patterns between the two intersecting genotypes. (1.71 MB TIF) [file ppat.1000988.s004.tif]

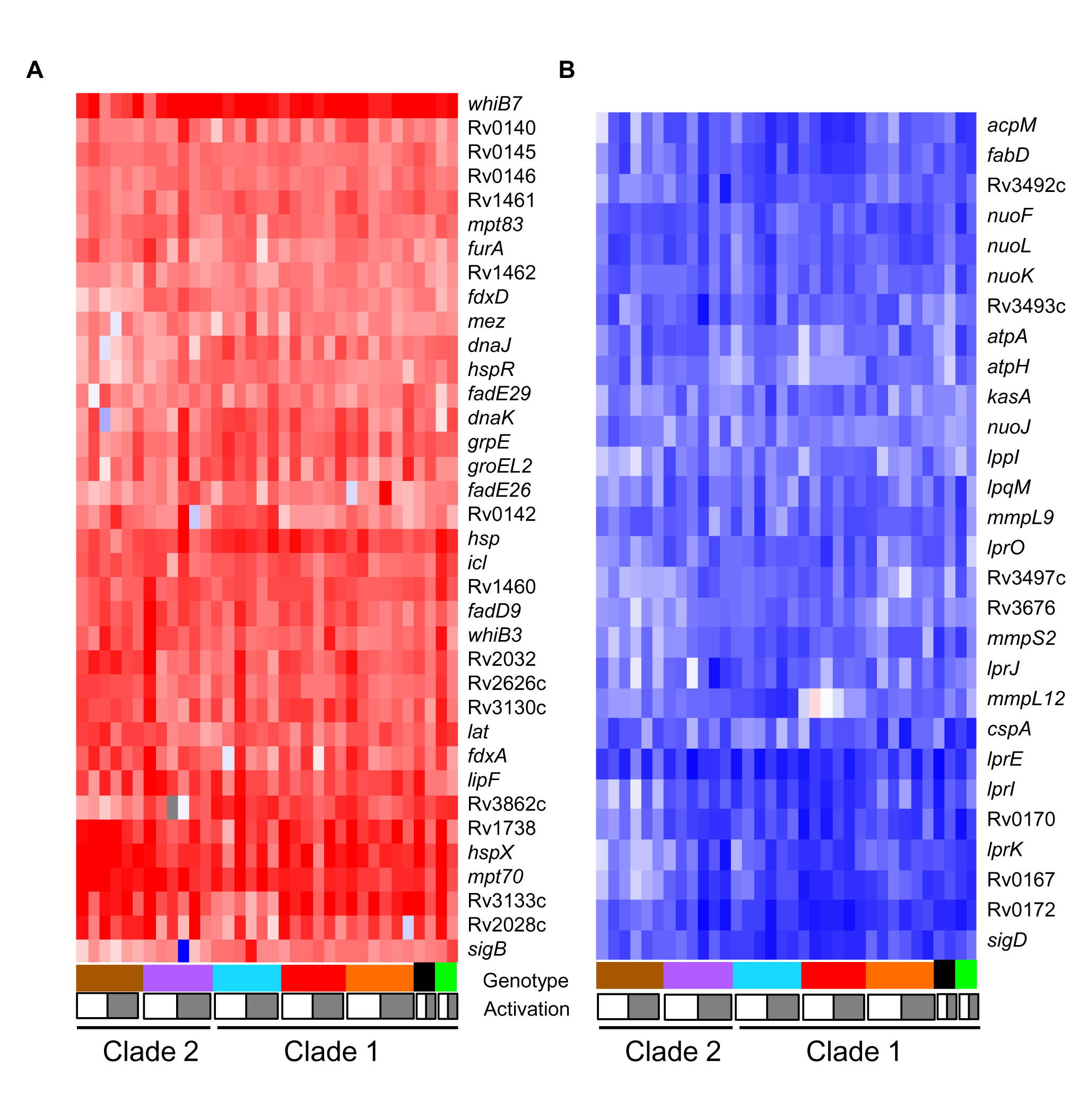

Supplement: Figure S5 — Genes displaying conserved induction (A) or repression (B) in both resting and activated macrophage phagosomes (24h post-infection) across our panel of MTC clinical isolates. Raw data were derived from array analysis of 17 MTC strains comparing intracellular transcript levels to extracellular controls of the same strain. “Universal” genes were selected as detailed in Figure 4 legend, with a subset of genes shown here. Genotypes are indicated by the color bar at bottom, which corresponds to color code shown in Figure 1A. Black indicated CDC1551 and green indicates H37Rv. Samples from both resting (white) and activated (gray) macrophage were included. (A) Universally induced genes included members of the DosR dormancy regulon (Rv2032, Rv2626c, Rv3130c, Rv1738, hspX, Rv3133c, Rv2028c), pH stimulon (lipF), ROI stress response (furA, fdxD, Rv1460-1461, fdxA), general stress (dnaJ, hspR, dnaK, grpE, groEL2, hsp), regulation (whiB7, furA, sigB, whiB6 (Rv3862c)), and lipid metabolism (mez, icl, fadD9). (B) Universally repressed genes included Type I NADH dehydrogenase (nuo), ATP synthase (atp), mycolic acid synthesis (acpM, fabD, kasA, transcriptional regulators (i.e. cspA, sigD, Rv3676), putative lipid transporters mce1 (Rv0167, Rv0171, Rv0172), mce4 (Rv3492c, Rv3493c, Rv3497c), mmpL9, mmpS2, mmpL12, and numerous lipoproteins (lppL, lpqM, lprO, lprJ, lprE, lprL, lprK). (1.96 MB TIF) [file ppat.1000988.s005.tif]

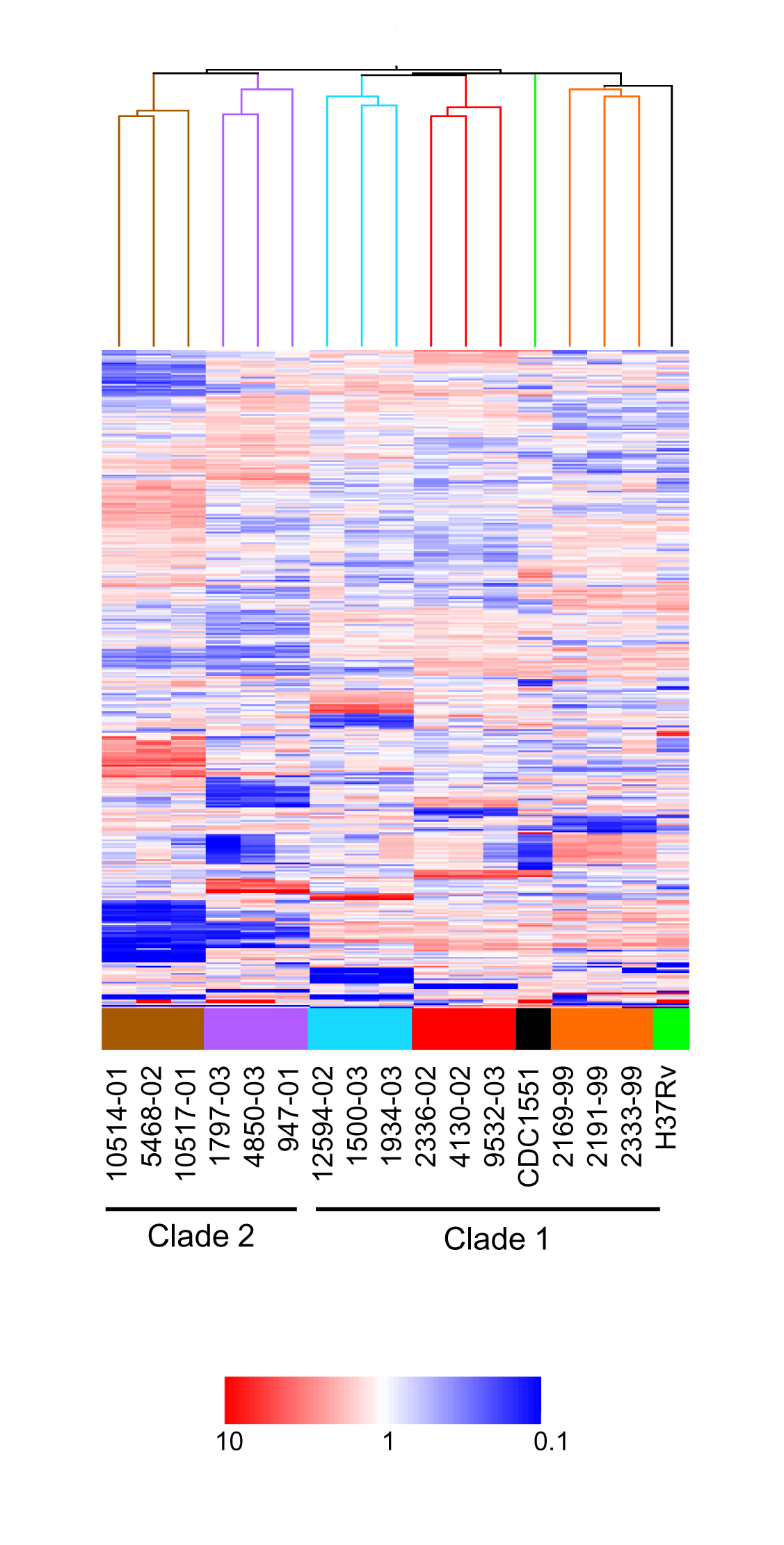

Supplement: Figure S6 — Direct comparison of transcript levels from intracellular MTC isolates after 24h infection of resting macrophages. Microarray images were loaded into Genespring as a single color experiment and normalized against the median. This effectively assesses the additive effect of baseline in vitro differences and intracellular changes in gene expression. Condition tree (Spearman correlation) of clinical isolates based on 499 genes determined by one-way ANOVA to exhibit genotype-dependent profiles (using Benjamini and Hochberg False Discovery Rate p<0.01). Compare with phylogenetic tree (Fig. 1A) and note clustering of strains according to genotype and delineation of clade 1 and clade 2 strains based on “absolute” intracellular gene expression. (0.80 MB TIF) [file ppat.1000988.s006.tif]

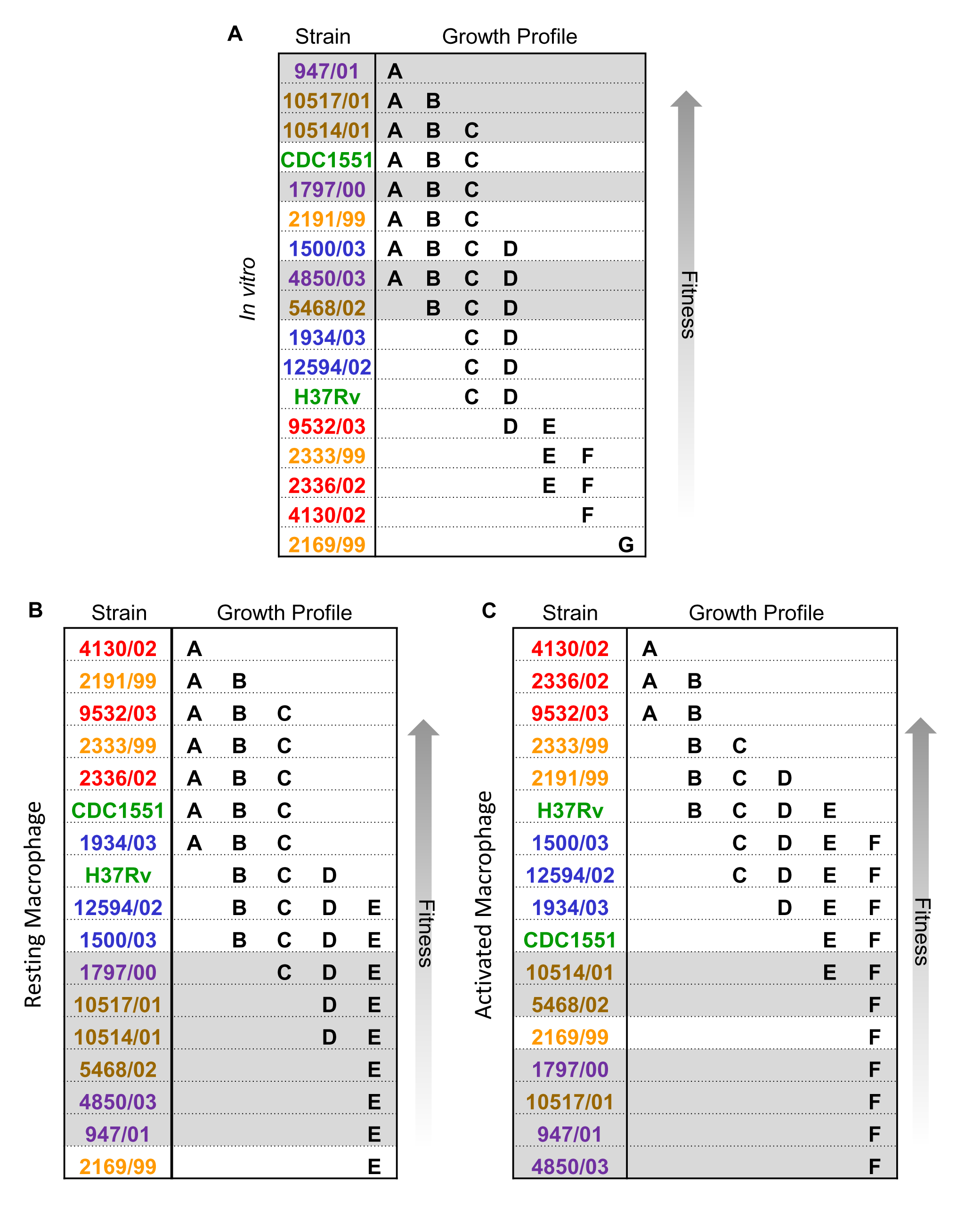

Supplement: Figure S7 — MTC clinical isolates display significantly different growth and survival profiles in vitro and in murine macrophages. The results of ANOVA and pair-wise comparisons between strains using the Tukey-Kramer HSD are summarized in connecting letter reports. Strains that do not share a letter (A–G in growth profile column) are significantly different (p<0.05). In A), for example, 2169/99 is significantly different from all other strains whereas 4130/02 is similar to 2336/02 and 2333/99. Strains are ranked from top to bottom by least-squares differences means which correlates with the growth/fitness of strains in vitro (A), in resting macrophages (B), or activated macrophages (C). Strain names are color coded to indicate genotype (red = Haarlem, blue = Beijing, orange = Uganda, purple = EAI, brown = West African 2). Clinical isolates belonging to clade 1 are shown with a white background while clade 2 isolates are highlighted in gray. (1.88 MB TIF) [file ppat.1000988.s007.tif]

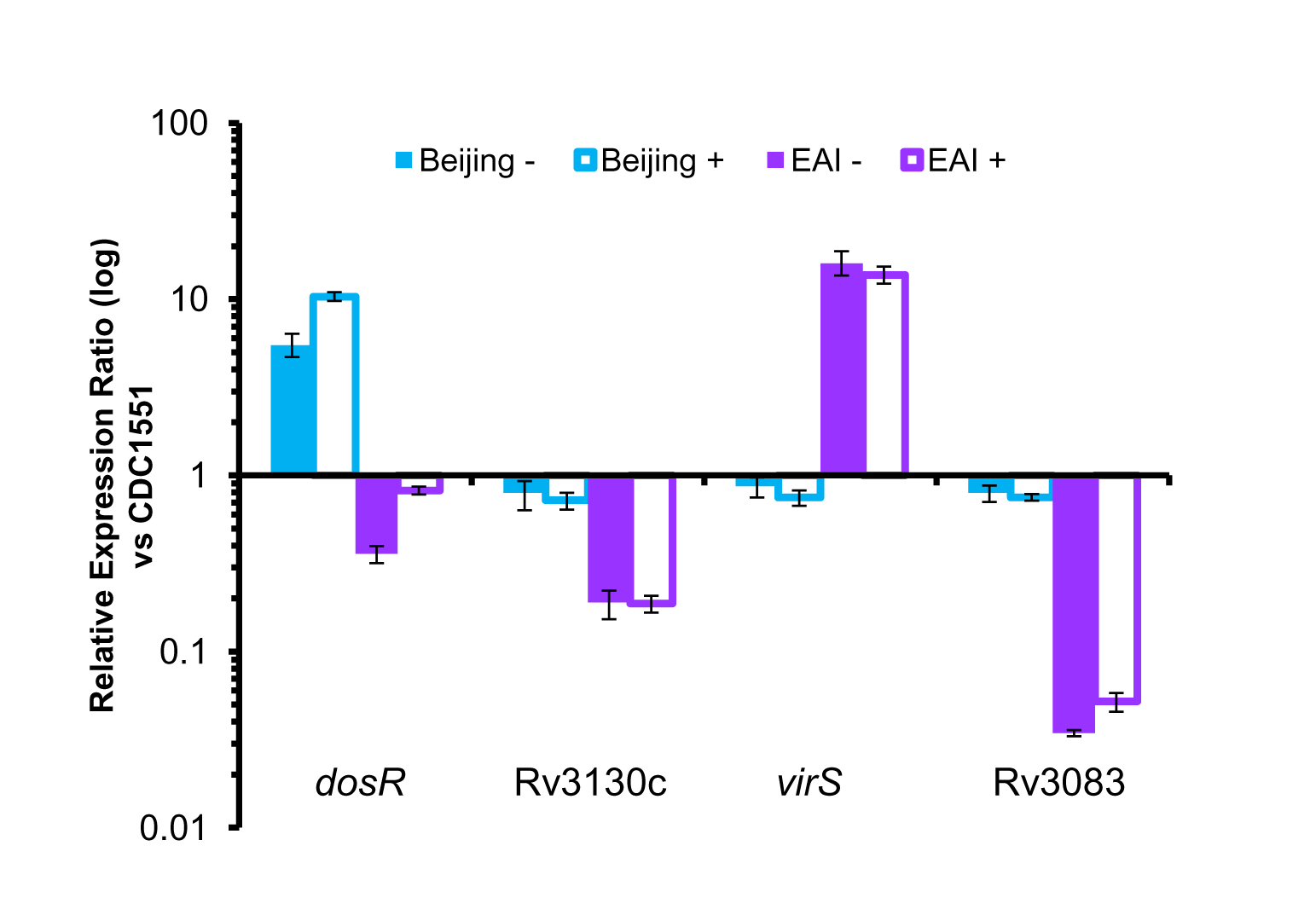

Supplement: Figure S8 — Validation of linear RNA amplification by qRT-PCR. Unamplified and amplified RNA from log phase in vitro mycobacteria including CDC1551 (reference strain), Beijing strain 12594/02, and EAI strain 4850/03 was reverse transcribed and quantified by relative qRT-PCR by normalization to sigA. Expression ratios shown are relative to the reference strain CDC1551. Solid bars and open bars represent the use of unamplified and amplified RNA, respectively. There was an excellent correlation between expression ratios derived from amplified and unamplified RNA. Note the overexpression of the dosR two-component response regulator in the Beijing strain. Although the dosR-dependent gene Rv3130c is expressed higher in Beijing versus EAI, transcript levels are comparable between 12594/02 and the reference strain, CDC1551. The overexpression of virS and concomitant repression of Rv3083 in EAI versus Beijing and CDC1551 verifies the genotype-specific regulation of this operon. Error bars indicate the standard deviation of ΔΔCT values calculated as described in the Guide to Performing Relative Quantitation of Gene Expression Using Real-Time Quantitative PCR (ABI). (0.35 MB TIF) [file ppat.1000988.s008.tif]

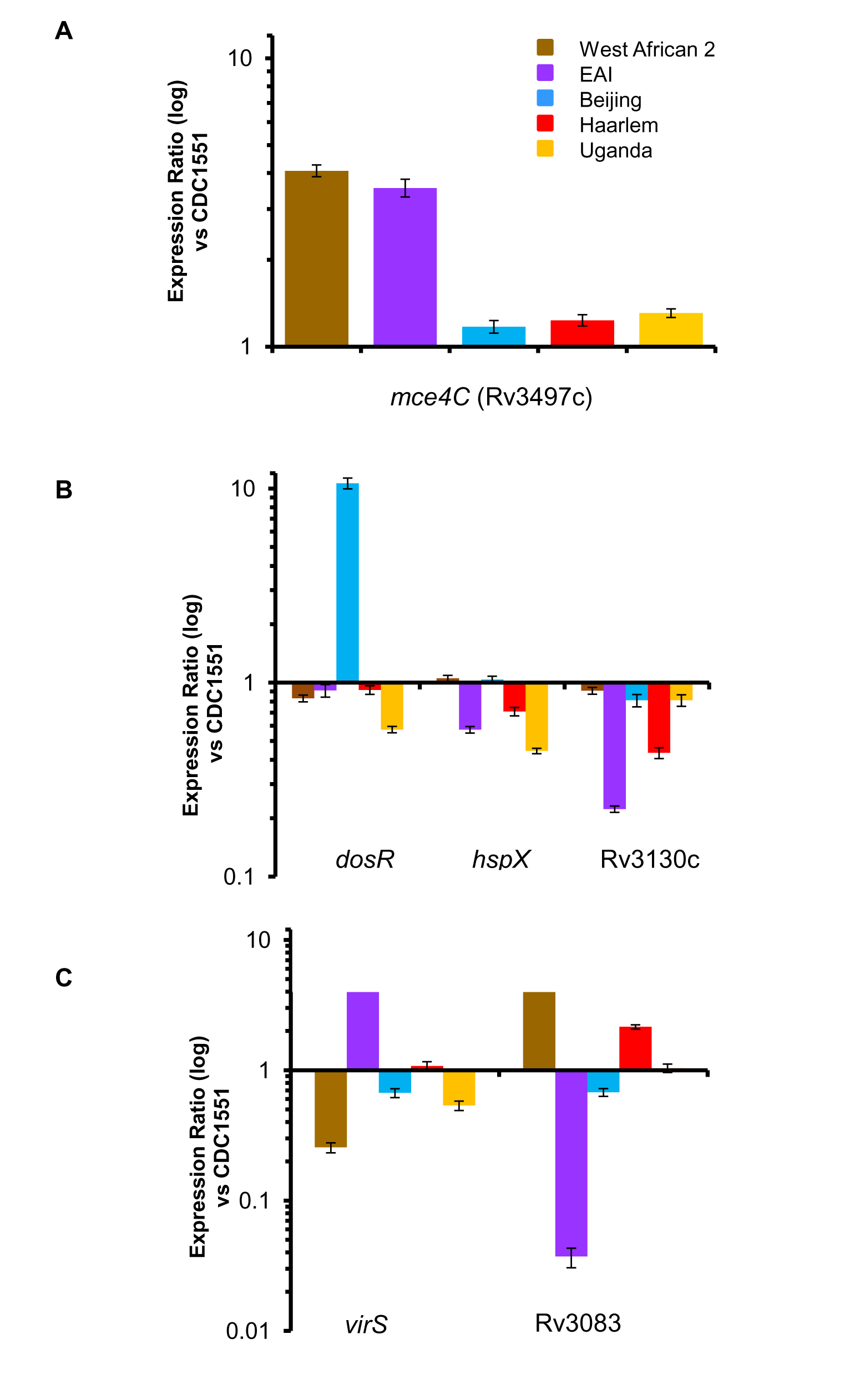

Supplement: Figure S9 — qRT-PCR analysis of select mycobacterial virulence factors exhibiting strain-dependent expression profiles in vitro. A) Overexpression of mce4C (involved in cholesterol uptake and metabolism) in West African 2 and EAI (clade 2 strains). B) Beijing genotype specific overexpression of dosR (response regulator of the hypoxia/dormancy regulon) but not dosR-dependent effectors (hspX, Rv3130c). C) EAI genotype specific overexpression of virS transcriptional regulator and repression of virS-dependent Rv3083. The color legend shown in A), which corresponds to colors used in all figures to designate specific genotypes, was also applied in B) and C). See legend for Supplementary Figure S8 for description of error bars. (0.70 MB TIF) [file ppat.1000988.s009.tif]

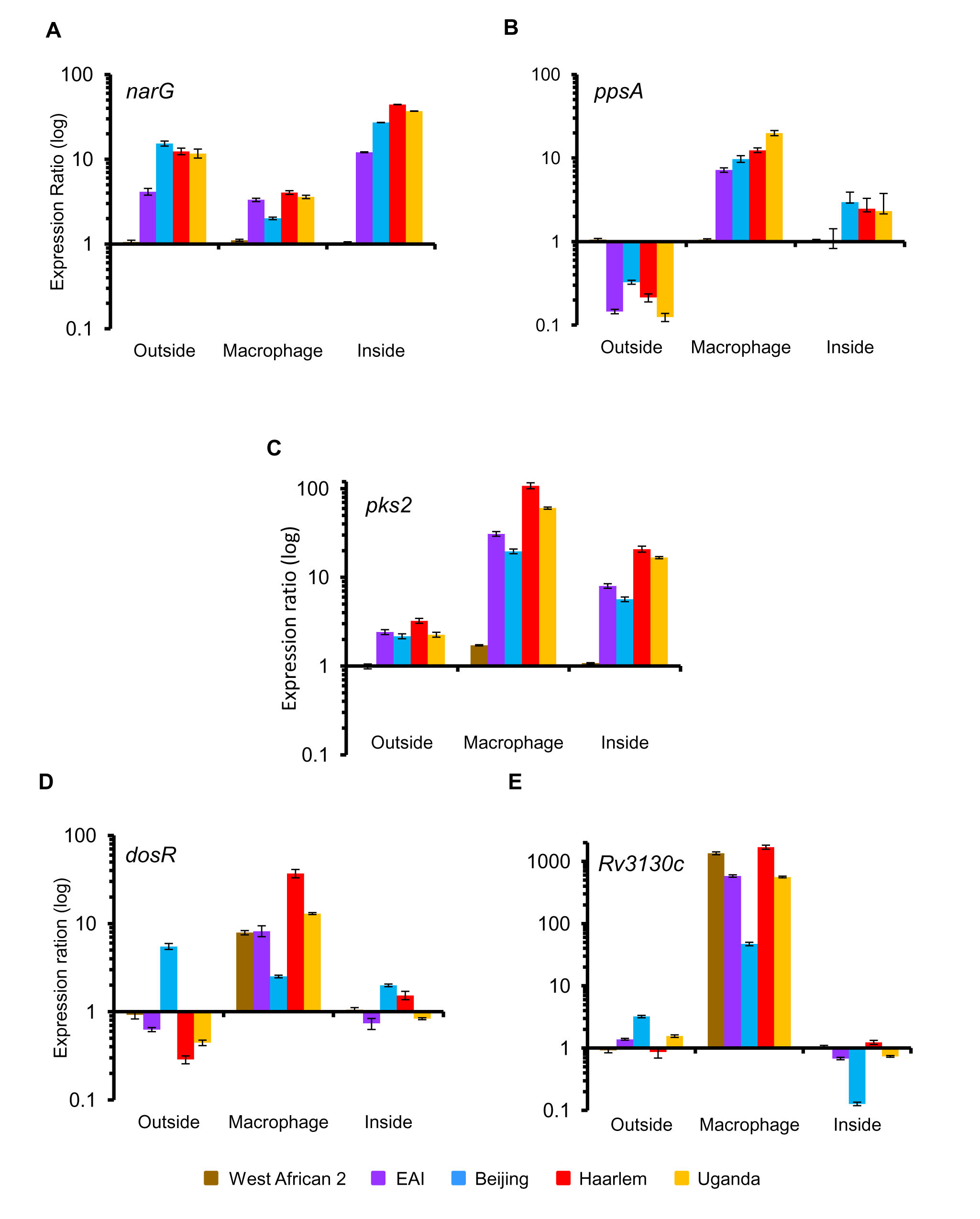

Supplement: Figure S10 — qRT-PCR analysis of select mycobacterial virulence factors exhibiting strain-dependent expression profiles during intracellular growth within macrophages. Data labeled “Outside” at the left of each panel represents the direct comparison (as detailed in Materials and Methods) of transcript levels in macrophage-controls incubated in DMEM infection medium for 24hr at 37C. Similarly, data labeled “Inside” at the right of each panel reflects direct comparison of transcript levels from intracellular MTC 24hr post-infection. For each gene, expression ratios “outside” and “inside” are expressed relative to West African 2 following normalization to sigA from the same sample. “Macrophage” expression ratios represent changes of gene expression upon macrophage invasion relative to levels in extracellular, macrophage-free controls. A) The underexpression of narG in vitro (outside) and lack of intracellular induction (macrophage) in West African 2 strains leads to large differences in transcript levels within the phagosome (inside). B) Differential basal expression and intracellular induction of ppsA in West African 2 strains. C) Elevated expression of dosR by extracellular control Beijing strains coupled with dampened induction upon macrophage infection results in comparable levels of dosR in the phagosome across all genotypes. D) Intracellular transcript levels of the dosR-dependent triacylglycerol synthase Rv3130c were lowest in the Beijing genotype, reflecting the ∼10-fold lower induction inside macrophages. E) Reduced transcript levels of pks2, a polyketide synthase involved in synthesis of the cell wall component sulfolipid [32], [34], by West African 2 strains is a combined result of lower baseline expression in vitro and defective upregulation within the phagosome. See legend for Supplementary Figure S8 for description of error bars. (1.03 MB TIF) [file ppat.1000988.s010.tif]
